# Gelsolin alleviates rheumatoid arthritis by negatively regulating NLRP3 inflammasome activation

**DOI:** 10.1038/s41418-024-01367-6

**Published:** 2024-08-24

**Authors:** Jiyeon Lee, Fumiyuki Sasaki, Eri Koike, Minjeong Cho, Yeongun Lee, So Hee Dho, Jina Lee, Eunji Lee, Eri Toyohara, Mika Sunakawa, Mariko Ishibashi, Huynh Hiep Hung, Saki Nishioka, Ritsuko Komine, Chiaki Okura, Masumi Shimizu, Masahito Ikawa, Akihiko Yoshimura, Rimpei Morita, Lark Kyun Kim

**Affiliations:** 1 Department of Biomedical Sciences, Graduate School of Medical Science, Brain Korea 21 Project, Gangnam Severance Hospital, Yonsei University College of Medicine, Seoul, Republic of Korea; 2 Department of Microbiology and Immunology, Nippon Medical School, Tokyo, Japan; 3 Immunology Frontier Research Center, Osaka University, Suita, Japan; 4 Department of Microbiology and Immunology, Keio University School of Medicine, Tokyo, Japan

**Keywords:** Immune cell death, Diagnostic markers, Rheumatic diseases

## Abstract

Despite numerous biomarkers being proposed for rheumatoid arthritis (RA), a gap remains in our understanding of their mechanisms of action. In this study, we discovered a novel role for gelsolin (GSN), an actin-binding protein whose levels are notably reduced in the plasma of RA patients. We elucidated that GSN is a key regulator of NLRP3 inflammasome activation in macrophages, providing a plausible explanation for the decreased secretion of GSN in RA patients. We found that GSN interacts with NLRP3 in LPS-primed macrophages, hence modulating the formation of the NLRP3 inflammasome complex. Reducing GSN expression significantly enhanced NLRP3 inflammasome activation. GSN impeded NLRP3 translocation to the mitochondria; it contributed to the maintenance of intracellular calcium equilibrium and mitochondrial stability. This maintenance is crucial for controlling the inflammatory response associated with RA. Furthermore, the exacerbation of arthritic symptoms in GSN-deficient mice indicates the potential of GSN as both a diagnostic biomarker and a therapeutic target. Moreover, not limited to RA models, GSN has demonstrated a protective function in diverse disease models associated with the NLRP3 inflammasome. Myeloid cell-specific GSN-knockout mice exhibited aggravated inflammatory responses in models of MSU-induced peritonitis, folic acid-induced acute tubular necrosis, and LPS-induced sepsis. These findings suggest novel therapeutic approaches that modulate GSN activity, offering promise for more effective management of RA and a broader spectrum of inflammatory conditions.

## Introduction

Rheumatoid arthritis (RA) is a chronic autoimmune disease characterized by persistent inflammation in synovial joints, leading to progressive cartilage degeneration and bone erosion [1]. In developed countries, RA prevalence ranges from 0.5 to 1.0% and is projected to increase with the rising aging population [2]. A multitude of risk factors have been implicated in RA, and early detection is pivotal for disease management and effective intervention. Hence, the identification of reliable diagnostic and prognostic biomarkers remains imperative. To date, numerous factors have been found to influence the disease trajectory; however, the precise pathophysiological mechanisms underlying the contribution of certain proposed biomarkers to disease progression remain unclear.

Gelsolin (GSN) is a protein with six conserved homologous domains. It primarily acts as an actin-binding protein. It regulates actin dynamics by severing and capping filamentous actin (F-actin) and sequestering actin monomers (G-actin) [3, 4]. GSN predominantly occurs in two isoforms: plasma GSN (pGSN) and cytoplasmic GSN (cGSN). pGSN differs from cGSN by the presence of a signal peptide, causing it to be secreted into the extracellular space. Moreover, pGSN contains an additional 24 amino acid residues at the N-terminal compared with that of cGSN [3]. Notably, pGSN acts as a biomarker for RA [5, 6]. The low pGSN level in the plasma of patients with RA may be associated with the consumption of circulating actin in inflamed joints [5] although this hypothesis requires further investigation. Moreover, both pGSN and cGSN are encoded by the same gene and undergo alternative splicing, despite the fact that the role of cGSN in the cells of patients with RA remains unknown. Thus, understanding the roles of these two proteins in RA pathogenesis is crucial for determining the effectiveness of pGSN as a predictive marker.

Numerous studies have reported the involvement of the NLRP3 inflammasome in RA pathogenesis [7, 8]. Canonical inflammasomes are multicomponent protein complexes that play key roles in inflammatory responses by activating caspase-1, which cleaves pro-interleukin (IL)-1β and the pore-forming protein gasdermin D (GSDMD). This leads to the secretion of mature IL-1β and pyroptosis [9, 10]. IL-1β contributes to synovial inflammation and cartilage degeneration in RA [8]. Both patients with RA and experimental arthritis mouse models exhibit elevated IL-1β and NLRP3 levels [7, 8]. Furthermore, treatment with the NLRP3 inhibitor MCC950 alleviates the severity of arthritis in mouse models [7], indicating the pivotal role of the NLRP3 inflammasome in RA pathogenesis. The NLRP3 inflammasome activation process has been studied in the context of its role in various intracellular organelles such as mitochondria, mitochondria-associated ER membranes (MAMs), and the Golgi apparatus [11–15]. Recent studies indicate that the complex ultimately forms at the microtubule-organizing center (MTOC) because active caspase-1 and IL-1β co-localize with the MTOC during inflammasome assembly [16]. Despite these advances, few studies have explored the location of NLRP3 during priming, and molecules that regulate NLRP3 subcellular localization during this process are not well understood. Potentially, excessive inflammasome activation can be effectively prevented by controlling NLRP3 expression or activity before secondary stimulation.

In this study, we demonstrate that GSN is a unique negative regulator of NLRP3 inflammasome activation. cGSN interacts with NLRP3 during the priming step that occurs prior to NLRP3 inflammasome assembly and activation and effectively prevents NLRP3 translocation to the mitochondria. This maintains intracellular calcium levels below the threshold at which the mitochondria are potentially damaged. However, the exacerbation of inflammation may increase the demand for cGSN to bind with NLRP3. As a result, pGSN may also have a chance to interact with NLRP3 intracellularly prior to its extracellular secretion. This intracellular binding could explain the reduced levels of pGSN observed in the plasma of patients with rheumatoid arthritis.

## Results

### cGSN binds to NLRP3 during the priming stage

**Fig. 1:**
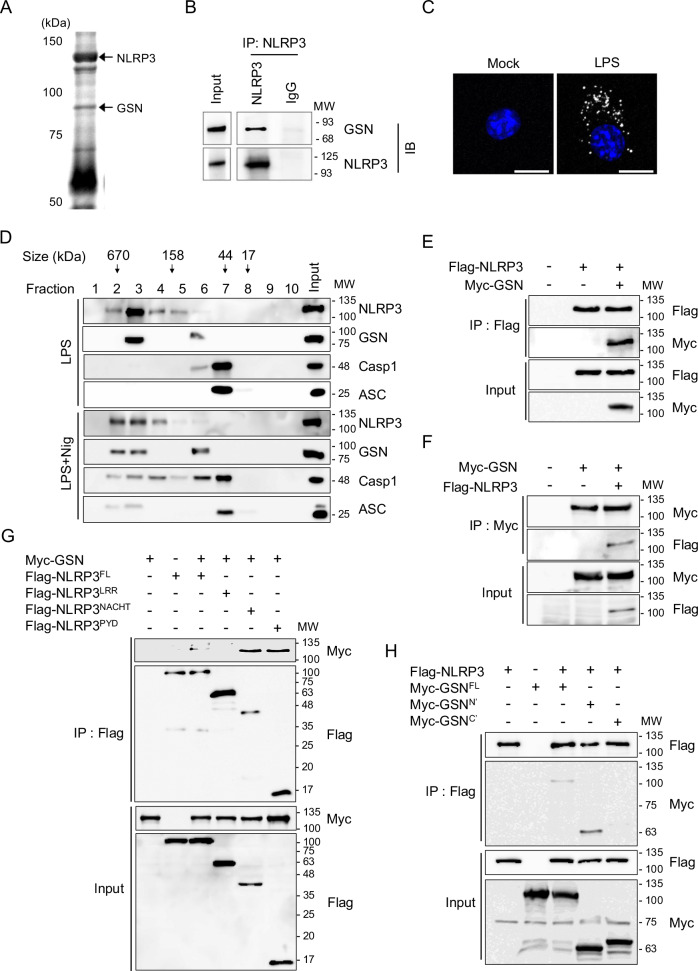
Gelsolin (GSN) interacts with NLRP3 in lipopolysaccharide (LPS)-primed macrophages. Representative images of an Oriole^®^-stained gel (**A**) and immunoblots of immunoprecipitates (**B**) after incubation of LPS (100 ng/mL, 3 h)-primed murine BMDMs with an anti-NLRP3 antibody. **C** In situ proximity ligation assay (PLA) of GSN–NLRP3 complexes in LPS (100 ng/mL, 3 h)-primed BMDMs. GSN–NLRP3 PLA signals, white; nuclei, blue. Scale bar: 10 μm. **D** Immunoblots of cell lysate in LPS (500 ng/mL, 3 h)-primed murine BMDMs stimulated with nigericin (5 μM, 45 min) and fractionated using gel-filtration chromatography. Immunoblots of co-immunoprecipitated proteins after incubation with an anti-Flag (**E**, **G**, **H**) or anti-Myc (**F**) antibody in HEK293T cells transiently transfected with Flag-NLRP3, Flag-NLRP3^LRR^, Flag-NLRP3^NACHT^, Flag-NLRP3^PYD^, Myc-GSN, Myc-GSN^N’^, and Myc-GSN^C’^. Data shown in (**B**, **C**, and **E**–**H**) are representative of at least three independent experiments.

We performed co-immunoprecipitation (Co-IP) using an anti-NLRP3 antibody in LPS-primed bone marrow-derived macrophages (BMDMs) to identify potential NLRP3 regulators that act prior to the assembly and activation of the NLRP3 inflammasome complex. Mass spectrometric analysis of the immunoprecipitates showed that cGSN is a binding partner of NLRP3 (Fig. 1A). This interaction was confirmed using endogenous Co-IP and proximity ligation assays (PLA) in LPS-primed BMDMs (Fig. 1B, C). Upon stimulation, NLRP3 oligomerized and recruited ASC to form an inflammasome complex with caspase-1 [9]. Following nigericin stimulation, NLRP3 expression shifted to a higher molecular mass fraction, indicating the formation of an NLRP3 inflammasome complex, as evidenced by gel filtration chromatography results (Fig. 1D). Notably, cGSN co-existed with NLRP3 in the LPS-primed BMDMs, whereas ASC and pro-caspase-1 co-eluted with NLRP3 after nigericin stimulation (Fig. 1D). Therefore, cGSN forms a complex with NLRP3 before ASC recruitment, which mediates the early stages of inflammasome assembly. Subsequently, NLRP3 and cGSN were transiently co-transfected into HEK293T cells to determine whether cGSN directly binds to NLRP3. Co-IP assays showed that cGSN co-eluted with NLRP3 (Fig. 1E, F) but not with other NLRP3 inflammasome components such as ASC, pro-caspase-1, or NEK7 (Fig. S1) [17, 18]. Thus, cGSN specifically interacts with NLRP3 during the priming stage prior to inflammasome assembly. Furthermore, to identify the NLRP3 regions that interacted with cGSN, we constructed various truncated mutants of these proteins. We found that the LRR domain of NLRP3 and the C-terminal of cGSN had defects in binding with each other; therefore, the NACHT and pyrin domains of NLRP3 and the N-terminal of cGSN were identified as essential mediators of this interaction (Fig. 1G, H).

### GSN negatively regulates NLRP3 inflammasome activation and pyroptosis

**Fig. 2:**
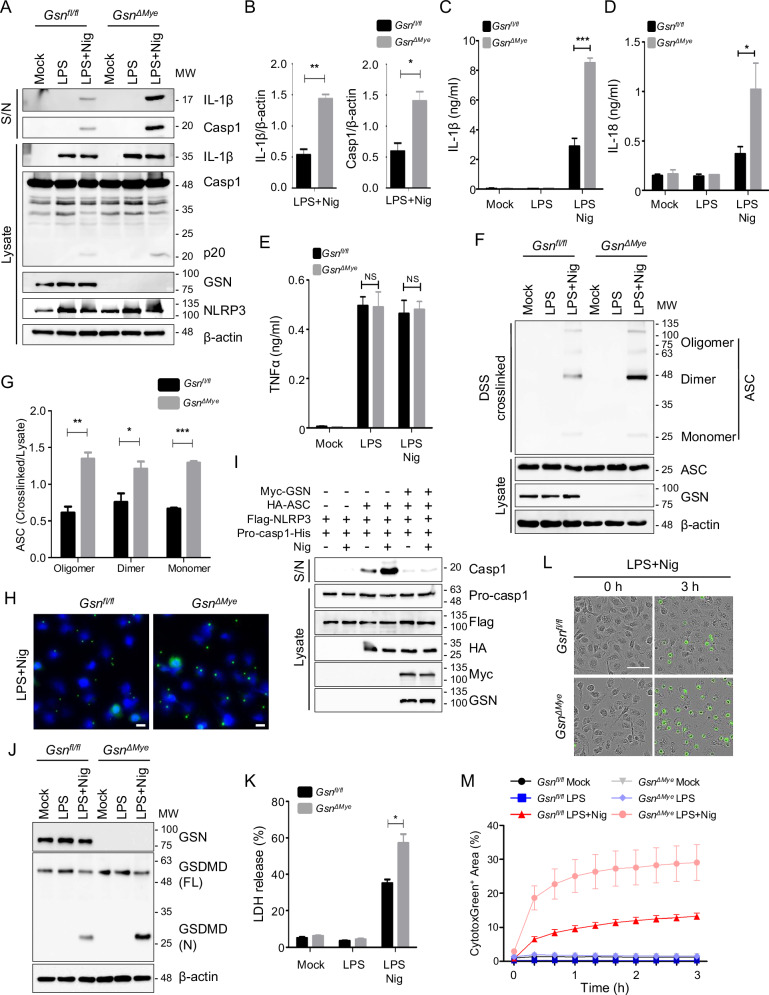
GSN deficiency results in excessive NLRP3 inflammasome activation and pyroptotic cell death. Immunoblots (**A**) and quantification (**B**) of supernatant (S/N) and cell lysates (Lysate) of *Gsn*^*fl/fl*^ and *Gsn*^*ΔMye*^ BMDMs left untreated, primed with LPS (500 ng/mL, 3 h), and primed and stimulated with nigericin (5 μM, 45 min). ELISA of IL-1β (**C**), IL-18 (**D**), and TNFα (**E**) in the supernatant of *Gsn*^*fl/fl*^ and *Gsn*^*ΔMye*^ BMDMs left untreated, primed with LPS (500 ng/mL, 3 h), and primed and stimulated with nigericin (5 μM, 45 min) (n = 3). NS, not significant. Immunoblots (**F**) and quantification (**G**) of ASC oligomerization of DSS cross-linked pellets and lysates of *Gsn*^*fl/fl*^ and *Gsn*^*ΔMye*^ BMDMs left untreated, primed with LPS (500 ng/mL, 3 h), and primed and stimulated with nigericin (5 μM, 45 min). **H** Immunofluorescence staining of ASC in LPS (100 ng/mL, 3 h)-primed murine BMDMs stimulated with nigericin (5 μM, 15 min). Nuclei were counterstained with Hoechst 34580. ASC, green; nuclei, blue. Scale bar: 10 μm. **I** Immunoblots S/N and lysate of HEK293T cells transiently transfected with Myc-GSN, HA-ASC, Flag-NLRP3, and Pro-casp1-His and stimulated with nigericin (20 μM, 45 min). **J** Immunoblots of full length and cleaved GSDMD in lysate of *Gsn*^*fl/fl*^ and *Gsn*^*ΔMye*^ BMDMs left untreated, primed with LPS (500 ng/mL, 3 h), and primed and stimulated with nigericin (5 μM, 45 min). **K** LDH release into the supernatants of *Gsn*^*fl/fl*^ and *Gsn*^*ΔMye*^ BMDMs left untreated, primed with LPS (500 ng/mL, 3 h), and primed and stimulated with nigericin (5 μM, 45 min) (n = 3). Representative IncuCyte images (**L**) and quantification (**M**) of CytotoxGreen^+^ dead cells in *Gsn*^*fl/fl*^ and *Gsn*^*ΔMye*^ BMDMs left untreated, primed with LPS (500 ng/mL, 3 h), and primed and stimulated with nigericin (5 μM, 45 min). Images were acquired at ×200 magnification. Scale bar: 50 μm (n = 6). Data are presented as mean ± SD (**C**–**E**) or mean ± SEM (**K**, **M**). Student’s *t* test, *P < 0.05, ***P < 0.001 (**B**, **C**, **I**). Data shown in (**A**, **F**, **H**, **I**, **J**) are representative of at least three independent experiments.

To elucidate the biological function of GSN in the NLRP3 inflammasome activation pathway, we generated a *Gsn*-deficient J774.1 cell clone and myeloid cell-specific *Gsn*-deleted (*Gsn*^*ΔMye*^) mice (Fig. S2A, B). Due to the deletion of the common region in cGSN and pGSN, the expression of both proteins is consequently reduced in these mice. No substantial differences were noted in the cellular composition between *Gsn*^*fl/fl*^ and *Gsn*^*Δmye*^ mice at the resting stage (Fig. S2C). To understand the role of GSN in the NLRP3 inflammasome pathway, we examined the nigericin-induced cleavage of pro-IL-1β and pro-caspase-1. *Gsn*^*Δmye*^ BMDMs exhibited excessive secretion of mature IL-1β (p17) and activated caspase-1 (p20) into the culture supernatant compared with that of the *Gsn*^*fl/fl*^ BMDMs (Fig. 2A–C). Moreover, the quantity of secreted IL-18, which is also released during NLRP3 inflammasome activation [19], was higher in the *Gsn*^*ΔMye*^ BMDMs than in the *Gsn*^*fl/fl*^ BMDMs (Fig. 2D); however, no effect was observed on the release of TNFα (Fig. 2E). Upon activation, the ASC undergoes oligomerization via homotypic PYD–PYD interactions [19]. We observed increased ASC oligomerization in *Gsn*^*ΔMye*^ BMDMs than in *Gsn*^*fl/fl*^ BMDMs (Fig. 2F, G). Additionally, gel filtration chromatography showed enhanced co-elution of ASC and caspase-1 with NLRP3 that shifted into a higher molecular weight fraction in *Gsn*^*ΔMye*^ BMDMs than in WT BMDMs (Fig. S3A and Fig. 1D). ASC oligomerization is visualized as a “speck” under the microscope, and we observed a significant increase in ASC specks in *Gsn*^*ΔMye*^ BMDMs than in *Gsn*^*fl/fl*^ BMDMs (Fig. 2H), indicating that the absence of GSN promoted NLRP3 inflammasome formation. Similar results were observed in nigericin-stimulated *Gsn*-KO J774.1 cells (Fig. S3B–H) and *Gsn*^*ΔMye*^ peritoneal-resident macrophages (Fig. S3I, J). Moreover, another NLRP3 inflammasome activator, imiquimod, induced higher IL-1β secretion and caspase-1 and -11 activation in *Gsn*^*ΔMye*^ peritoneal-resident macrophages than those observed in *Gsn*^*fl/fl*^ peritoneal-resident macrophages (Fig. S4). However, GSN deficiency had no effect on the accumulation of acetylated-α-tubulin, which mediates microtubule-dependent inflammasome activation in control and *Gsn*-KO J774.1 cells (Fig. S3B). The NLRP3 inflammasome reconstitution system in HEK293T cells, which was used to further clarify the inhibitory effects of GSN on NLRP3 inflammasome activation, showed that the overexpression of NLRP3, ASC, and pro-caspase-1 in endogenous inflammasome protein-deficient HEK293T cells triggered pro-caspase-1 maturation upon nigericin stimulation [20, 21]. As expected, cGSN overexpression inhibited nigericin-induced caspase-1 cleavage (Fig. 2I). Moreover, GSN deficiency did not affect the cytosolic LPS- or flagellin-induced activation of noncanonical- or NLRC4 inflammasomes, respectively. However, GSN may be involved in poly(dA:dT) transduction-induced AIM2 inflammasome activation (Fig. S5A–C). Additionally, GSN did not influence LPS-induced nuclear factor κB (NF-κB) activation, as evidenced by comparable levels of NF-κB activation and mRNA expression of *Nlrp3*, *Casp1*, and *Il1b* in *Gsn*^*ΔMye*^ and *Gsn*^*fl/fl*^ BMDMs (Fig. S5D–G). As expected, the mRNA expression of *Tnf* did not differ (Fig. S5H).

Furthermore, the absence of GSN increased NLRP3 inflammasome-dependent pyroptotic cell death, as evidenced by the generation of the GSDMD N-terminal fragment (GSDMD-N) and the release of lactate dehydrogenase (LDH) (Fig. 2J, K and Fig. S6). Additionally, live-cell imaging showed that a higher number of dead cells were stained with cytotoxin green in the absence of GSN (Fig. 2L, M). Overall, these findings show that GSN specifically inhibits NLRP3 inflammasome activation and caspase-1-mediated pyroptosis.

### GSN inhibits NLRP3 translocation to mitochondria during priming process by disturbing the NLRP3 and MAVS associations

In macrophages, NLRP3 expression is initiated by a primary stimulus such as LPS and subsequently forms a complex with ASC and pro-caspase-1 upon a secondary stimulus like nigericin. Extensive research has been conducted to determine the intracellular localization of NLRP3 at each stage. Some studies suggest that NLRP3 associates with MAMs [13] or the Golgi apparatus [14, 20], particularly with the dispersed trans-Golgi network (dTGN) [15]. For the formation of the inflammasome complex, NLRP3 associates with ASC on the mitochondria at the perinuclear region in a microtubule-dependent manner [11, 12]. Ultimately, the NLRP3 inflammasome is assembled and activated at the MTOC [16, 22, 23]. Although the exact subcellular localization of NLRP3 at each stage is still debated, mitochondrial translocation of NLRP3 is one of the crucial initial steps in NLRP3 inflammasome activation, as LPS priming alone is sufficient to induce the association of NLRP3 with mitochondria [24].

**Fig. 3:**
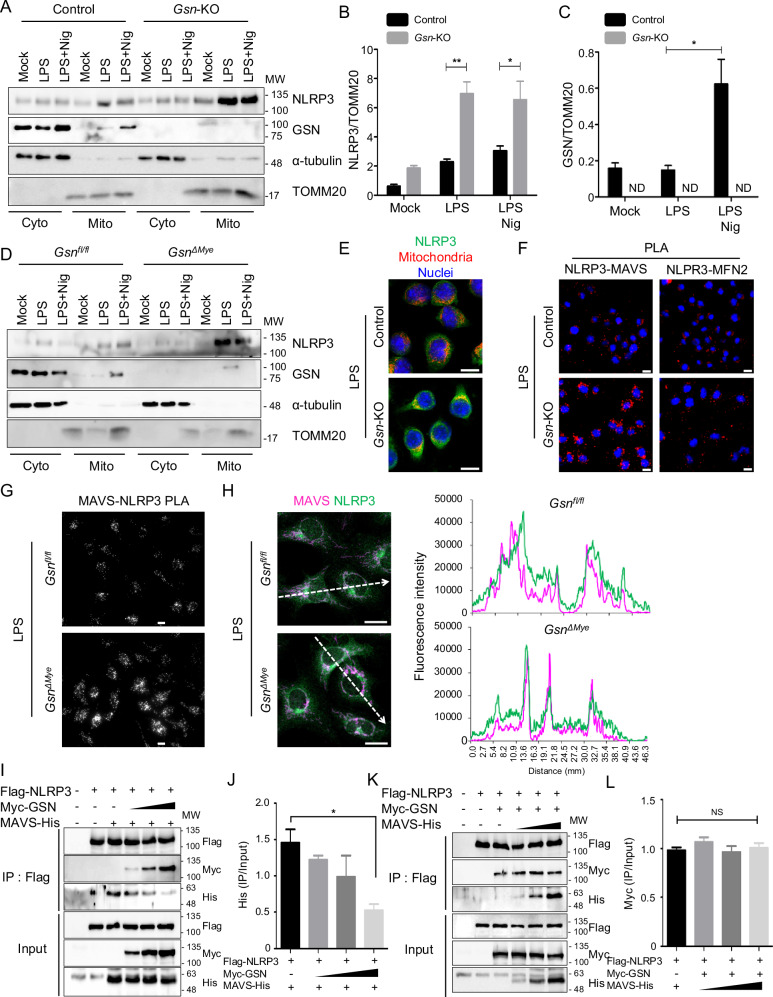
GSN depletion promotes NLRP3 translocation to mitochondria. **A**–**C** Immunoblots of the cytosolic (Cyto) and mitochondrial (Mito) fractions in control and *Gsn*-KO J774.1 cell left untreated, primed with LPS (500 ng/mL, 3 h), and primed and stimulated with nigericin (5 μM, 45 min). The intensity of immunoblot bands was quantified using the ImageJ software (**B**, **C**) (n = 4). ND, not detected. **D** Immunoblots of Cyto and Mito fractions in *Gsn*^*fl/fl*^ and *Gsn*^*ΔMye*^ BMDMs left untreated, primed with LPS (500 ng/mL, 3 h), and primed and stimulated with nigericin (5 μM, 45 min). **E** Immunostaining of NLRP3 and labeling of mitochondria and nuclei in control and LPS (100 ng/mL, 3 h)-primed *Gsn*-KO J774.1 cells. **F** In situ PLA of NLRP3-MAVS and NLRP3-MFN2 complexes in control and LPS (100 ng/mL, 3 h)-primed *Gsn*-KO J774.1 cells. PLA signals, red; nuclei, blue. **G** In situ PLA of NLRP3-MAVS complexes in *Gsn*^*fl/fl*^ and *Gsn*^*ΔMye*^ peritoneal resident macrophages primed with LPS (100 ng/mL, 3 h). PLA signals, white. **H** Immunofluorescence of co-staining of MAVS and NLRP3 in *Gsn*^*fl/fl*^ and *Gsn*^*ΔMye*^ peritoneal resident macrophages primed with LPS (100 ng/mL, 3 h). The graphs on the right show the fluorescence intensity of MAVS (pink) and NLRP3 (green), as indicated by the white dotted line arrows in the photos on the left. **I**–**L** Immunoblots of co-immunoprecipitated proteins following incubation with an anti-Flag antibody in HEK293T cells transiently transfected with Myc-GSN, Flag-NLRP3, and MAVS-His. The intensity of immunoblot bands was quantified using the ImageJ software (**J**, **L**) (n = 3). NS, not significant. Data are presented as mean ± SEM (**B**, **C**, **J**, **L**). Student’s *t* test, *P < 0.05, **P < 0.01 (**B**, **C**, **J**). Data shown in (**A**, **E**–**I**, **K**) are representative of at least three independent experiments. Scale bar; 10 μm (**E**–**H**).

As cGSN interacts with NLRP3 during the LPS-priming stage, we decided to investigate how GSN affects the intracellular localization of NLRP3, focusing initially on its translocation to the mitochondria. To investigate this, we isolated cytosolic and mitochondrial fractions and confirmed the presence of NLRP3 in the mitochondrial fraction during the priming stage (Fig. 3A, B). Notably, NLRP3 translocation to the mitochondrial fraction was higher in *Gsn*-KO J774.1 cells than in the control J774.1 cells, suggesting GSN mediated inhibition of NLRP3 movement to this organelle (Fig. 3A, B). Furthermore, cGSN itself translocated to the mitochondrial fraction upon nigericin stimulation, presumably in its free form without interacting with NLRP3 during the LPS-priming step (Fig. 3A, C). Similar results were obtained in *Gsn*^*ΔMye*^ BMDMs (Fig. 3D). Immunocytochemistry further corroborated these findings by showing that NLRP3 co-localized with mitochondria upon LPS priming and that this co-localization was enhanced during GSN deficiency (Fig. 3E).

NLRP3 inflammasome stimuli cause the mitochondrial outer membrane protein MAVS to interact with NLRP3, which facilitates NLRP3 translocation to the mitochondria[11]. Another mitochondrial outer membrane protein, MFN2, also interacts with NLRP3 and guides it to the mitochondria following RNA virus infection [25]. Therefore, we investigated whether GSN affected the interaction between NLRP3 and these mitochondrial proteins. We observed enhanced PLA signals for the NLRP3–MAVS complex but not for the NLRP3–MFN2 complex in LPS-primed *Gsn*-KO J774.1 cells compared with those in control cells; this suggests that cGSN inhibits the interaction between NLRP3 and MAVS (Fig. 3F). Similar results were observed in LPS-primed *Gsn*^*Δmye*^ peritoneal-resident macrophages compared with those in *Gsn*^*fl/fl*^ macrophages (Fig. 3G). Additionally, the co-localization of NLRP3 and MAVS was enhanced in LPS-primed *Gsn*^*Δmye*^ peritoneal resident macrophages compared with that in *Gsn*^*fl/fl*^ macrophages (Fig. 3H). Further investigation of these results using Co-IP experiments showed that NLRP3 and MAVS were co-transfected with varying quantities of cGSN. The interaction between NLRP3 and MAVS gradually decreased as the cGSN level increased. providing confirmation that cGSN competes with MAVS for the interaction with NLRP3 (Fig. 3I, J). However, co-transfection of NLRP3 and cGSN with varying concentrations of MAVS did not affect the interaction between NLRP3 and cGSN, indicating that cGSN may exert a higher affinity than MAVS towards NLRP3 (Fig. 3K, L). Collectively, these findings indicate that cGSN prevent NLRP3 from being translocated to the mitochondria by interfering with the NLRP3–MAVS interaction.

Lastly, we decided to examine the effect of GSN on the ER localization of NLRP3. We observed that NLRP3 is also located in the ER, as well as in the mitochondria, during the priming stage. Interestingly, while the ER is distributed throughout the cytoplasm in *Gsn*^*fl/fl*^ peritoneal-resident macrophages, it is primarily located in the perinuclear region in *Gsn*^*Δmye*^ cells. This suggests that the increased translocation of NLRP3 to the mitochondria in the perinuclear region may be due to this ER localization pattern in *Gsn*^*Δmye*^ cells (Fig. S7).

### GSN acts as a calcium reservoir and maintains mitochondrial stability

**Fig. 4:**
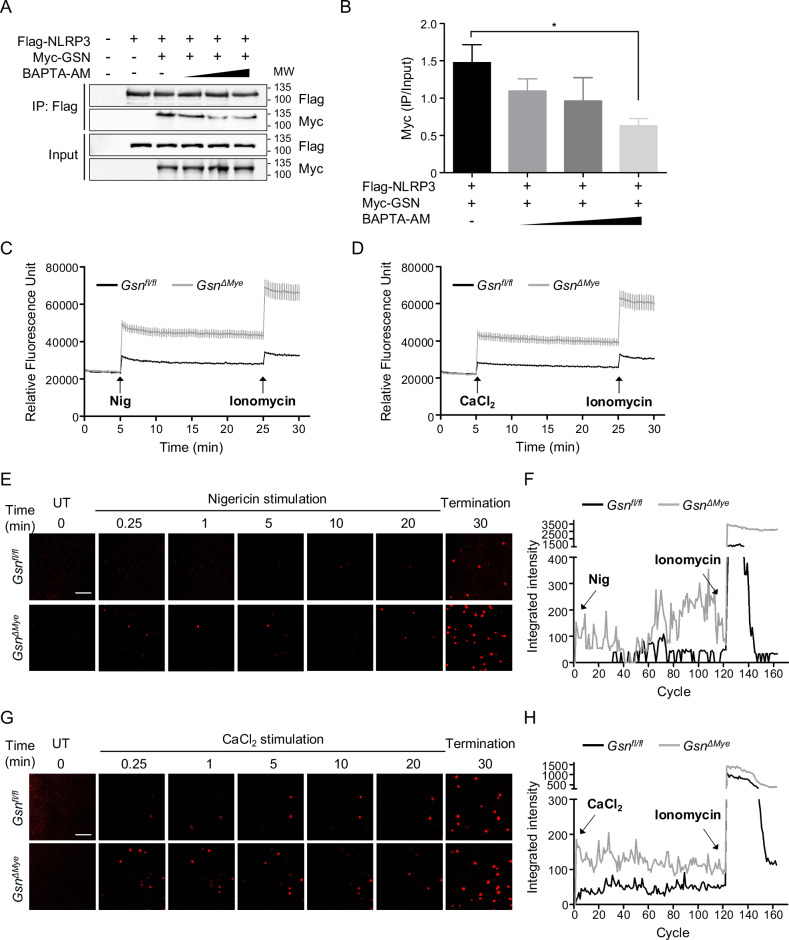
Intracellular calcium concentration increased in the absence of GSN. **A**, **B** Immunoblots of co-immunoprecipitated proteins after incubation with an anti-Flag antibody in HEK293T cells transiently transfected with Myc-GSN and Flag-NLRP3 in the presence or absence of BAPTA-AM (**A**). The intensity of immunoblot bands was quantified using the ImageJ software (**B**) (n = 3). Calcium influx in LPS-primed peritoneal resident macrophages in response to nigericin (20 μM) (**C**) or CaCl_2_ (1 mM) (**D**) stimulation was analyzed using a microplate reader. Ca^2+^ ionophore ionomycin (5 μM) was added after 25 min (n = 10). Representative confocal microscopy images (**E**, **G**) and quantification (**F**, **H**) of calcium influx in LPS-primed peritoneal resident macrophages in response to nigericin (20 μM) (**E**, **F**) or CaCl_2_ (1 mM) (**G**, **H**) stimulation. Ca^2+^ ionophore ionomycin (5 μM) was added after 30 min, and cells were further imaged for 10 min. Images were acquired at ×200 magnification. Scale bar: 50 μm. Data are presented as mean ± SEM (**B**–**D**). Student’s *t* test, *P < 0.05 (**B**). Data shown in (**A**) are representative of at least three independent experiments.

GSN is activated by various factors, including intracellular calcium [3]. We investigated whether GSN activity influences the NLRP3–GSN interaction. Chelation of intracellular calcium by BAPTA-AM reduced the interaction between NLRP3 and cGSN in a dose-dependent manner. Thus, calcium-activated cGSN binds to NLRP3 (Fig. 4A, B). GSN has multiple calcium-binding sites and effectively sequesters calcium ions upon binding [3]. As cGSN maintains calcium level under normal conditions for binding to NLRP3, we hypothesized that the free intracellular calcium concentration would be higher in GSN-deficient macrophages than in control macrophages. Moreover, NLRP3 inflammasome stimulation promotes calcium mobilization [26]. In this study, nigericin and CaCl_2_ stimulation triggered an influx of calcium into the cytosol (Fig. 4C, D). This influx was significantly promoted in the *Gsn*^*Δmye*^ peritoneal macrophages. These results were corroborated and visualized microscopically (Fig. 4E–H).

**Fig. 5:**
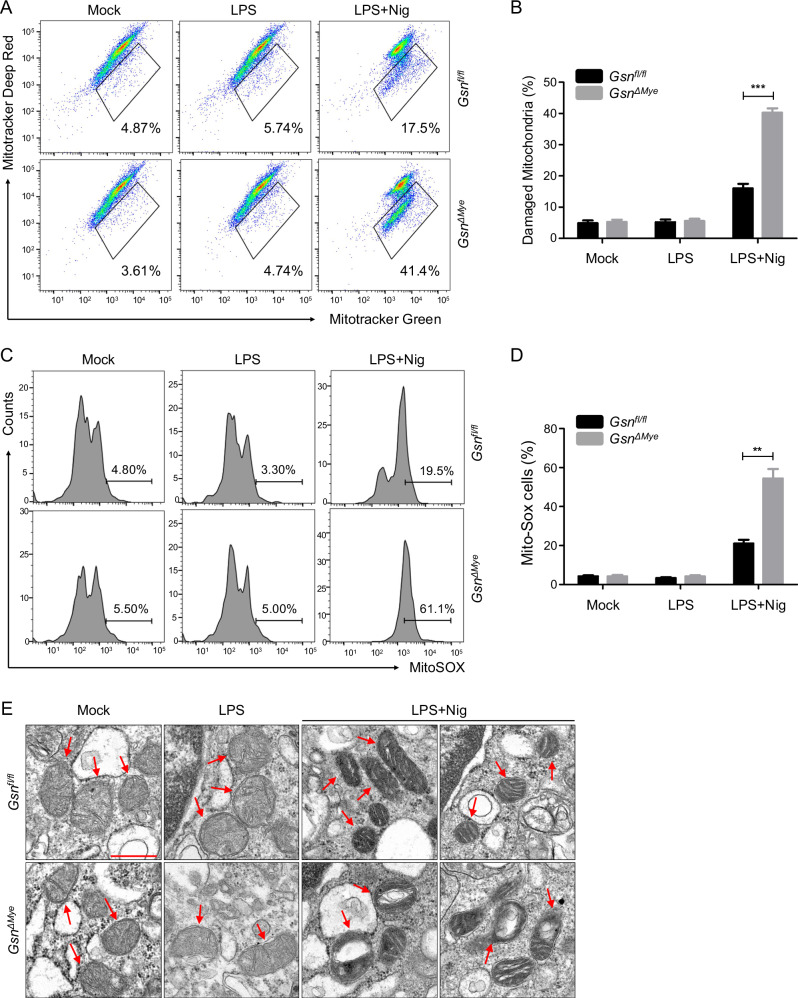
GSN ablation promotes mitochondrial dysfunction. Representative dot plots (**A**) and histograms (**C**) of flow cytometry and quantification (**B**, **D**) of damaged mitochondria (surrounded by black lines in **A**) (n = 4) and MitoSOX^+^ mitochondria (indicated by black bars in C) (n = 3) in *Gsn*^*fl/fl*^ and *Gsn*^*ΔMye*^ BMDMs left untreated, primed with LPS (500 ng/mL, 3 h), and primed and stimulated with nigericin (10 μM, 1 h) (**A**, **B**) or (20 μM, 45 min) (**C**, **D**). **E** Representative TEM images of mitochondria from *Gsn*^*fl/fl*^ and *Gsn*^*ΔMye*^ peritoneal resident macrophages left untreated, primed with LPS (500 ng/mL, 3 h), and primed and stimulated with nigericin (5 μM, 45 min). To avoid bias, at least 10 images were acquired per sample. Red arrows indicate mitochondria. The images were acquired at ×72.0 k magnification. Scale bar: 5 μm. Data are presented as mean ± SEM (**B**, **D**). Student’s *t* test, **P < 0.01, ***P < 0.001 (**B**, **D**). Data shown in (**A**, **C**) are representative of at least three independent experiments.

Calcium signaling is associated with NLRP3 inflammasome activation, which is triggered by an increase in cytosolic calcium levels as a result of extracellular calcium treatment or calcium release from the ER [27]. Elevated intracellular calcium levels lead to calcium uptake by mitochondria, causing calcium overload and mitochondrial destabilization. Damaged mitochondria produce mROS, which activates the NLRP3 inflammasome [13, 28]. Mitochondrial damage is assessed by the loss of mitochondrial membrane potential (ΔΨm) [28]. We found that GSN depletion led to excessive ΔΨm loss, as evidenced by decreased mitochondrial respiration (Fig. 5A, B). Consistent with these results, mROS generation was promoted in *Gsn*^*Δmye*^ BMDMs rather than in *Gsn*^*fl/f*l^ BMDMs (Fig. 5C, D). Similar results were obtained for *Gsn*-KO J774.1 cells (Fig. S8A–D). The presence of electron-dense structures in TEM images of internal mitochondrial structures indicates mitochondrial structural defects [29]. Exposure to nigericin facilitated the accumulation of electron-dense mitochondria; this effect was highly exacerbated in the absence of GSN, as evidenced by the highly disrupted cristae (Fig. 5E). In *Gsn*^*Δmye*^ peritoneal macrophages, ruptured lysosomes were observed more frequently compared to *Gsn*^*fl/f*l^ peritoneal macrophages (Fig. S8E). These findings suggest that GSN acts as a calcium reservoir that binds to NLRP3, preventing NLRP3 inflammasome activation induced by mitochondrial damage.

### Reduced pGSN levels in RA are attributed to its protective function in inflammatory conditions

**Fig. 6:**
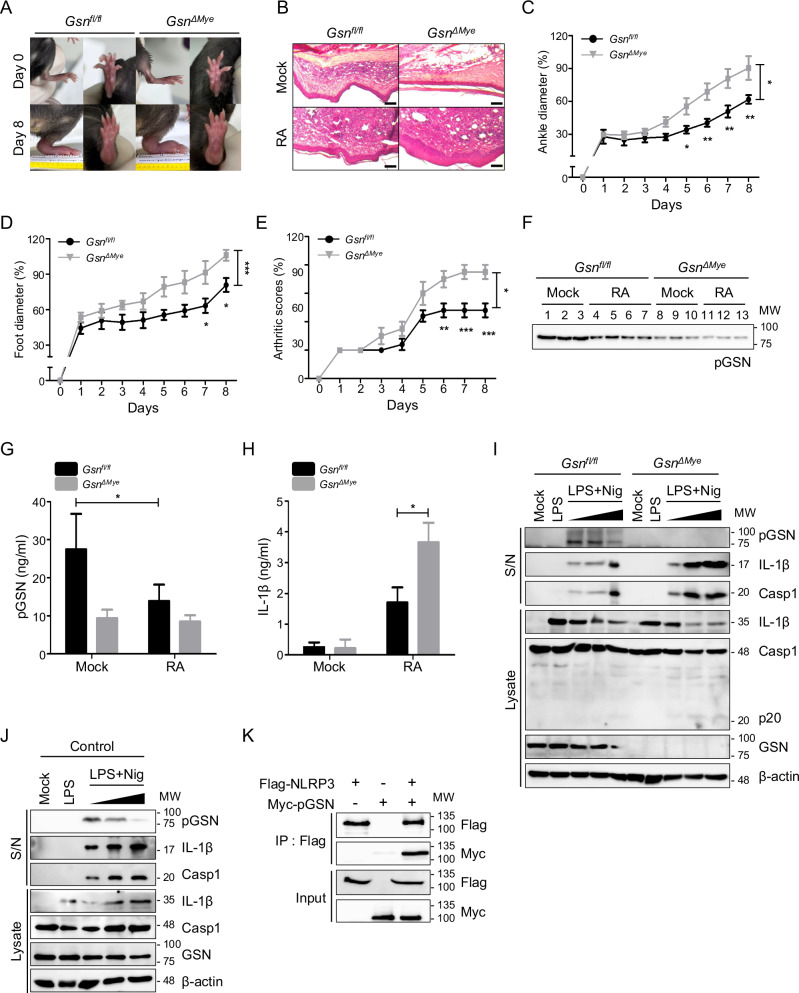
Extracellular GSN secretion decreases in rheumatoid arthritis, and GSN deficiency exacerbates the condition. **A** Representative images of the paws of *Gsn*^*fl/fl*^ and *Gsn*^*ΔMye*^ mice that were intraperitoneally injected with complete Freund’s adjuvant. **B** Representative hematoxylin and eosin-stained images of the paws of *Gsn*^*fl/fl*^ and *Gsn*^*ΔMye*^ mice at day 8 after injection with complete Freund’s adjuvant or incomplete Freund’s adjuvant. Scale bar: 200 μm. **C**–**E** Joint swelling was assessed by measuring ankle (**C**) and foot pad (**D**) thickness. **E** The severity of arthritis was graded on a scale of 0–4. **F** Immunoblots for pGSN in the plasma of *Gsn*^*fl/fl*^ and *Gsn*^*ΔMye*^ mice that were intraperitoneally injected with complete Freund’s adjuvant or incomplete Freund’s adjuvant. ELISA of pGSN (**G**) and IL-1β (**H**) in the plasma of *Gsn*^*fl/fl*^ and *Gsn*^*ΔMye*^ mice that were intraperitoneally injected with complete Freund’s adjuvant or incomplete Freund’s adjuvant. **I** Immunoblots of the supernatant (S/N) and cell lysates (Lysate) of *Gsn*^*fl/fl*^ and *Gsn*^*ΔMye*^ BMDMs left untreated, primed with LPS (500 ng/mL, 3 h), and primed and stimulated with nigericin (2 μM, 5 μM, and 20 μM for 45 min). **J** Immunoblots of supernatant (S/N) and cell lysates (Lysate) of control J774.1 cells left untreated, primed with LPS (500 ng/mL, 3 h), and primed and stimulated with nigericin (2 μM, 5 μM, and 20 μM for 45 min). **K** Immunoblots of co-immunoprecipitated proteins after subjecting HEK293T cells that were transiently transfected with Flag-NLRP3 and Myc-pGSN to incubation with an anti-Flag antibody. Data are presented as mean ± SEM (**C**–**E**) or mean ± SD (**G**, **H**). n = 3–4 (**A**–**H**). Two-way ANOVA followed by additional validation using Bonferroni method (**C**–**E**). Student’s *t* test (**G**, **H**). *P < 0.05 **P < 0.01 ***P < 0.001. Data shown in (**K**) are representative of at least three independent experiments.

To explore the underlying causes for the low plasma pGSN levels among patients with RA, we induced RA in *Gsn*^*Δmye*^ mice that have impaired pGSN and cGSN production in myeloid cells [5, 6]. The *Gsn*^*Δmye*^ mice exhibited exacerbated swelling in the ankle and foot compared with that of control mice, which is indicative of a significantly heightened inflammatory state (Fig. 6A–D). This led to overall arthritic scores (Fig. 6E). Immunoblotting and ELISA results confirmed that the pGSN levels in the plasma of control mice were as low as those observed in human patients on induction of the RA (Fig. 6F, G). However, pGSN was detected even in KO mice, suggesting the presence of pGSN sources apart from myeloid cells, with muscle cells being likely candidates [30]. As expected, the IL-1β levels increased, suggesting that inflammation was excessively induced in *Gsn*^*Δmye*^ mice because of inflammasome overactivation (Fig. 6H). Furthermore, the secretion of pGSN in both BMDM and J774.1 cells upon stimulation with LPS and nigericin was confirmed, and this quantity decreased as the treatment concentration increased (Fig. 6I, J). This reduction in pGSN concentration in plasma can be attributed to diminished secretion from myeloid cells. As both pGSN and cGSN are transcribed from a single gene and distinguished by alternative splicing, we examined the nanopore long-read sequencing data to distinguish between these two GSN isoforms in macrophages (Fig. S9A). Our findings showed the distinct presence of both types in macrophages. cGSN was considerably more abundant than pGSN; however, their expression did not significantly increase upon LPS treatment (Fig. S9B). Additionally, we verified that extracellularly detected GSN predominantly consisted of pGSN, whereas cGSN was not secreted even through exosomes (Fig. S9C). Thus, we speculate that the decrease in the quantity of pGSN secreted outside the cell as inflammation escalates may be attributed to its intracellular binding with NLRP3. This speculation was supported by the results of co-IP experiments, which confirmed the interaction between pGSN and NLRP3 (Fig. 6K).

### GSN inhibits NLRP3 inflammasome activation in non-RA in vivo models

**Fig. 7:**
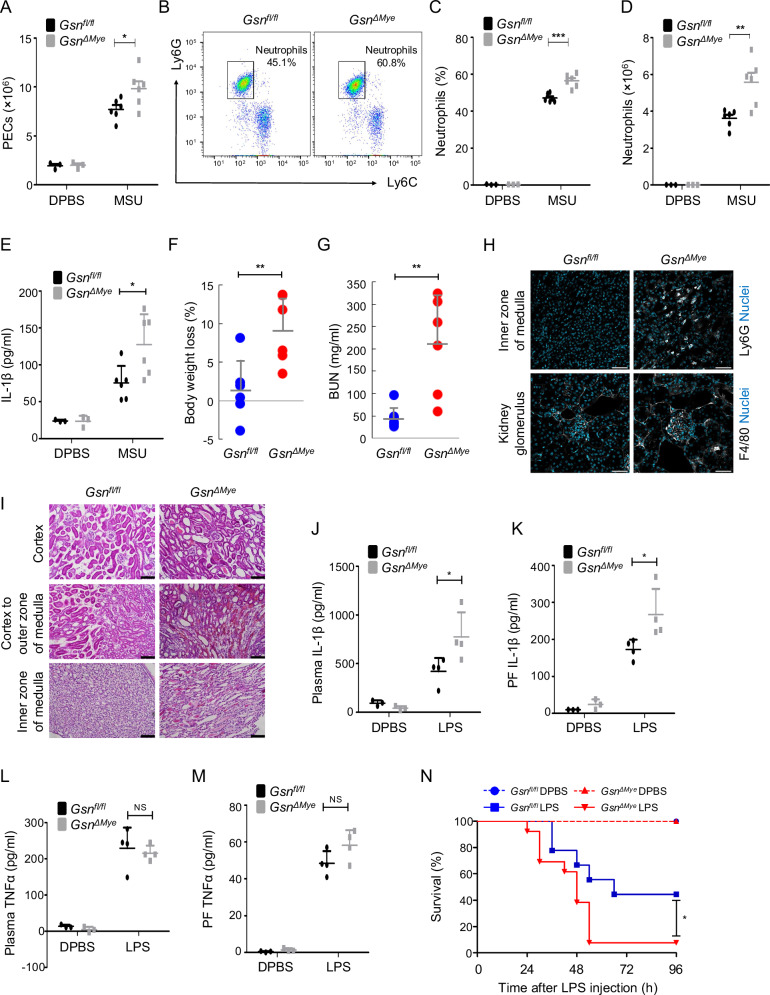
GSN deficiency aggravates immunopathologic responses in vivo. **A** Quantification of infiltrated peritoneal exudate cells (PECs) in *Gsn*^*fl/fl*^ and *Gsn*^*ΔMye*^ mice that were intraperitoneally injected with MSU crystals or sterile PBS. Representative dot plots of flow cytometry (**B**); quantification of the percentage (**C**) and number (**D**) of infiltrated neutrophils (CD11b^+^Ly6G^+^Ly6C^lo^ cells) in the peritoneal cavities of *Gsn*^*fl/fl*^ and *Gsn*^*ΔMye*^ mice that were intraperitoneally injected with MSU crystals or sterile PBS. **E** ELISA for IL-1β in the peritoneal lavage fluid of *Gsn*^*fl/fl*^ and *Gsn*^*ΔMye*^ mice that were intraperitoneally injected with MSU crystals or sterile PBS. Percentage of body weight loss (**F**) and blood urea nitrogen (BUN) concentration in the sera (**G**) of *Gsn*^*fl/fl*^ and *Gsn*^*ΔMye*^ mice that were intraperitoneally injected with folic acid (n = 6 mice per group). **H** Representative images of immunofluorescence from the kidneys of *Gsn*^*fl/fl*^ and *Gsn*^*ΔMye*^ mice at 40 h after folic acid treatment. Scale bar: 50 μm. **I** Representative hematoxylin and eosin-stained images of kidneys from *Gsn*^*fl/fl*^ and *Gsn*^*ΔMye*^ mice at 40 h after folic acid treatment. Scale bar: 20 μm. ELISA for plasma IL-1β (**J**), peritoneal lavage fluid (PF) IL-1β (**K**), plasma TNFα (**L**), peritoneal lavage fluid (PF) TNFα (**M**) of *Gsn*^*fl/fl*^ and *Gsn*^*ΔMye*^ mice that were intraperitoneally injected with 20 mg/kg LPS or sterile PBS (n = 3–4). NS, not significant. **N** Survival of *Gsn*^*fl/fl*^ (n = 12) and *Gsn*^*ΔMye*^ (n = 16) mice that were intraperitoneally injected with 10 mg/kg LPS or sterile PBS (n = 3 for each of *Gsn*^*fl/fl*^ and *Gsn*^*ΔMye*^). Data are presented as mean ± SEM (**A**, **C**, **D**) or mean ± SD (**E**–**G**, **J**–**M**). n = 3–6 (**A**–**E**). Student’s *t* test, *P < 0.05 **P < 0.01, ***P < 0.001 (**A**, **C**–**G**, **J**, **K**). Log-rank test, *P < 0.05 (**N**).

We used three mouse models to investigate whether GSN influences other inflammatory diseases involving NLRP3 inflammasome activation besides RA. MSU crystals, which cause gout, induce neutrophil infiltration and IL-1β secretion in an NLRP3 inflammasome-dependent manner when directly injected into the peritoneum [31, 32]. As expected, intraperitoneal administration of MSU crystals resulted in increased total PEC recruitment, neutrophil (CD11b^+^Ly6G^+^Ly6C^lo^) influx, and IL-1β release (Fig. 7A–E). These effects were more pronounced in *GSN*^*Δmye*^ mice and conformed with our in vitro results (Fig. 7A–E). Folic acid-induced acute tubular necrosis (ATN) is another mouse model of NLRP3-dependent inflammation [11, 33]. *Gsn*^*Δmye*^ mice exhibited greater weight loss and higher BUN levels than those of *Gsn*^*fl/fl*^ mice, which is indicative of kidney damage (Fig. 7F, G). The recruitment of neutrophils (Ly6G^+^ cells) and macrophages (F4/80^+^ cells) to the kidney was enhanced in the absence of GSN (Fig. 7H), and histopathological examination showed that pathological features such as tubular dilation and extracellular matrix deposits were more severe in *Gsn*^*Δmye*^ mice (Fig. 7I). IL-1β production following intraperitoneal administration of LPS is also dependent on NLRP3 [34]. After LPS challenge, IL-1β levels increased in both plasma and peritoneal lavage fluid of *Gsn*^*Δmye*^ mice compared with those of *Gsn*^*fl/fl*^ mice (Fig. 7J, K). However, TNFα levels did not differ significantly between the two groups in either plasma or peritoneal lavage fluid (Fig. 7L, M). Additionally, *Gsn*^*ΔMye*^ mice showed increased susceptibility to LPS-induced lethality than that of *Gsn*^*fl/fl*^ mice (Fig. 7N). These results further demonstrate the essential role of GSN in suppressing the in vivo NLRP3 inflammasome-dependent inflammatory response.

## Discussion

Recent years have seen an increase in active efforts to diagnose and monitor disease progression non-invasively. Although a plethora of markers have been proposed for various diseases based on observation, the scarcity of highly reliable markers can be attributed to the difficulty in identifying markers that collectively represent the diverse situations that patients face, including those arising from genetic or environmental differences. Additionally, the lack of clear elucidation regarding the actual roles of the proposed markers within the human body poses a challenge. Thus, a molecule, that plays significant roles in disease development within cells could be a reliable biomarker if it is secreted extracellularly, enabling non-invasive detection. Among the many diagnostic biomarkers that have been proposed for RA, pGSN could be an effective biomarker owing to its low levels that have been reported in the plasma and synovial fluid of patients with RA [5, 6]. Additionally, pGSN levels have been observed to decrease in mouse models of sepsis [35]. Nevertheless, the cellular roles of GSN and the reason for its low secreted levels in patients with RA have remained unknown.

In this study, we have confirmed that cGSN, which differs from pGSN by lacking a signal peptide, inhibits the formation of the NLRP3 inflammasome complex with ASC and pro-caspase-1, thereby regulating IL-1β production and pyroptosis-associated inflammation. The inflammasome activation process occurs in two steps. During the priming stage, NLRP3 is activated by stimuli such as LPS that trigger NF-κB before forming an inflammasome complex and becoming fully active [9, 10]. We initially hypothesized that understanding the mechanism underlying NLRP3 expression and regulation during this priming stage would help identify ways to suppress inflammasome activity regardless of the second stimulus. Thus, cGSN, which is a binding partner of NLRP3 during the priming stage, was identified as the study candidate.

There are many conflicting results regarding the subcellular localization of NLRP3. In macrophages, NLRP3 is transcriptionally induced during the priming step and forms the inflammasome complex upon secondary stimulation. However, its localization to mitochondria, ER, or other organelles during this process remains a topic of debate. This inconsistency may be due to the fact that most studies capture only a snapshot at a specific moment and are limited in that they focus on particular regions of interest rather than observing the entirety of NLRP3 proteins or the inflammasome complex. Our observations indicate that a portion of NLRP3 localizes to the mitochondria during the LPS priming step in macrophages. Additionally, some NLRP3 is also located in the ER. Interestingly, while the ER is generally distributed throughout the cytoplasm, in GSN-deficient macrophages, it is primarily localized in the perinuclear region. We also found that NLRP3 translocates more to the mitochondria in GSN-deficient macrophages, and that these mitochondria are predominantly located in the perinuclear region. Given that GSN is a protein that regulates actin dynamics, it is plausible that GSN could influence the intracellular positioning of organelles during the priming step through its interaction with NLRP3. However, as our study is also based on limited snapshots, further detailed study is required.

GSN exists in both cytosolic and plasma forms, despite neither form being transcribed individually; in fact, both forms are derived from alternative splicing of a single gene. Further studies are needed to elucidate the regulatory mechanisms governing alternative splicing. This study has shown that pGSN secretion from macrophages decreases as inflammation intensifies. We considered the role of GSN in intracellularly suppressing excessive inflammation and initially hypothesized that the reduction in secreted pGSN concentration correlates with its increased intracellular function. However, further investigation is required to verify this hypothesis. Additionally, a limitation of our study is that while we confirmed a decrease in pGSN secretion in the RA animal model, such a decrease was not significantly observed in other models including sepsis models and peritonitis models. It is uncertain whether this decrease in pGSN secretion is unique to RA among diseases where inflammasome activation is involved in pathogenesis, or if this observation was limited by the technical constraints of our study. Further precise and detailed research is also necessary to clarify this issue.

Nevertheless, despite these uncertainties, this study unequivocally demonstrates that GSN regulates NLRP3 inflammasome activation. This significant finding warrants further study of GSN, not only as a diagnostic biomarker for diseases like RA but also as a potential target for inflammation modulation.

## Materials and methods

### Mice

The floxed *Gsn* allele on mouse chromosome 2 was generated by Biotechnology Research and Development, Osaka University, using CRISPR-Cas9 gene editing, according to the following two-step method. Pronuclear-stage zygotes from C57/B6J mice were electroporated with an *EcoRV*-*loxP* site-containing single-stranded oligonucleotide (ssODN) and crRNA (AAACGTGATCTACCCTTCTGCAGCC); the latter targets intron 2 of *Gsn* and the Cas9 protein. Embryos were surgically transplanted into the oviducts of pseudo-pregnant ICR mice. Newborn mice bearing an *EcoRV*-*loxP* site in intron 2 were identified by genotyping. Pronuclear-stage zygotes from gene-modified mice were electroporated with a *loxP*-*XhoI* site-containing ssODN and another crRNA (CACCGAGGATCTCAGGATCGGGACT) that targets intron 3 of *Gsn* and the Cas9 protein. The second embryo was transplanted into pseudo-pregnant ICR mice, and newborn mice bearing both the *EcoRV*-*loxP* site in intron 2 and the *loxP*-*XhoI* site in intron 3 were identified through genotyping. Cre recombination resulted in the deletion of exon 3 of the floxed *Gsn* allele. *Gsn*^*fl/fl*^ mice were bred with C57/B6-*LysM*-*cre* mice (RBRC02302) to generate macrophage-specific *Gsn*-deficient mice (*Gsn*^*ΔMye*^) in a specific pathogen-free (SPF) animal facility at Nippon Medical School.

Mice were housed under SPF conditions. Mice (6–10 weeks old) from the same cage were randomly selected for different treatments. Littermate controls were included whenever possible. The experiments were performed on both females and males. The investigators were not blinded, as none of the reported experiments required subjective decision-making. Key experiments were repeated by at least three independent researchers. Primer sequences used for genotyping are listed in Supplementary Table S1.

### Cell culture

To generate mouse BMDMs, bone marrow cells were flushed from the femurs and tibias of 6–10-week-old mice using Dulbecco’s phosphate-buffered saline (DPBS). The cells were subsequently cultured in Dulbecco’s modified Eagle’s medium (DMEM) supplemented with 10% fetal bovine serum (FBS), 20% L929 cell-conditioned medium, and 100 U/mL of penicillin and streptomycin, respectively, for 7 days. To obtain mouse peritoneal-resident macrophages, the peritoneal exudate cells (PECs) were collected by injecting 10 mL of 5 mM ethylenediaminetetraacetic acid-containing PBS into the mouse peritoneal cavities at 6–10 weeks of age. The collected PECs were seeded into cell-culture plates or dishes. After 2 h, the non-attached cells were removed. The macrophages were cultured in DMEM supplemented with 10% FBS, 100 U/mL each of penicillin and streptomycin, and 2 mM L-glutamine. The J774.1 mouse macrophage cell line (RIKEN, Saitama, Japan) was cultured in Roswell Park Memorial Institute (RPMI) 1640 medium supplemented with 10% FBS, 1 mM sodium pyruvate, 2 mM L-glutamine, 100× MEM non-essential amino acids, 100 U/mL each of penicillin and streptomycin, and 50 μM 2-mercaptoethanol. The human embryonic kidney cell line HEK293T was cultured in DMEM supplemented with 10% FBS and 100 U/mL each of penicillin and streptomycin.

### Macrophage stimulation

To activate the NLRP3 inflammasome in macrophages, the cells were primed with 500 ng/mL LPS-EK Ultrapure (tlrl-peklps, Invivogen, San Diego, CA, USA) or 100 ng/mL LPS-EB Ultrapure from *Escherichia coli* 0111:B4 (tlrl-3pelps, Invivogen) in Opti-MEM (31985-070, Thermo Fisher Scientific, Waltham, MA, USA) for 3 h, followed by stimulation with the indicated concentrations of nigericin (tlrl-nig, Invivogen), imiquimod (1338313, Sigma, St. Louis, MO, USA), CPPD crystals (tlrl-cppd, Invivogen), MSU crystals (tlrl-msu, Invivogen), or hydroxyapatite (289396, Sigma) for the indicated time. To activate the AIM2 inflammasome, 0.75 µg/mL poly(dA:dT) (tlrl-patn, Invivogen) was transfected into LPS-primed cells using Lipofectamine 2000 (11668027, Invitrogen, Waltham, MA, USA). To activate the non-canonical inflammasome, 2 µg/mL ultrapure LPS from *E. coli* 0111:B4 was transfected into LPS-primed cells using FuGENE^®^ HD (E2311, Promega, Madison, WI, USA). To activate the NLRC4 inflammasome, 1.5 µg/mL flagellin from *Salmonella typhimurium* (SRP8029; Sigma) was transfected into LPS-primed cells using Lipofectamine 2000 (11668027; Invitrogen). After transfection, the cells were cultured for 2 h.

### CRISPR-Cas9 knockout cell clones

*Gsn*-deficient J774.1 cell clones were generated using CRISPR/Cas9 gene editing technology. The murine *Gsn* knockout target sites (ACACGTGGTACCCAATGAGGTGG, TCCCGCCAAACAAGCTCATGAGG, and CGCGCTCCACGTTGGCAATGTGG) were identified using the CHOPCHOP website (https://chopchop.cbu.uib.no). The sgRNA for each target site was synthesized using an in vitro transcription kit (EnGen^®^ sgRNA Synthesis Kit, *S. pyogenes* [E3322s, NEB, Ipswich, MA, USA]). After dephosphorylation using shrimp alkaline phosphatase (2660 A; Takara, Kusatsu, Japan), the sgRNA was purified using an RNA purification kit (Monarch^®^ RNA Cleanup Kit [T2040s; NEB]). Next, 500 ng of sgRNAs and 1.5 μg of Cas9 protein (M0646T, NEB) complexes were transfected into 1.7 × 10^5^ J774.1 cells using the TransIT-X2^®^ reagent (MIR6003, Mirus, Marietta, GA, USA). After 2 days, the cells were seeded into 96-well plates at a concentration of one cell per well. The *Gsn*-deficient J774.1 cell clone (P18-37) was selected for this study based on the western blotting analysis results. Parental J774.1 cells were used as controls.

### Protein identification

BMDMs were stimulated with LPS for 3 h and lysed in ice-cold cell lysis buffer (20 mM HEPES [pH 7.4], 150 mM NaCl, 1.5 mM MgCl_2,_ 10 mM KCl, 1 mM EDTA, 1 mM EGTA, 1 mM DTT, and 0.5% Nonidet P-40) containing protease and phosphatase inhibitor cocktails (Nacalai, Kyoto, Japan). After the cell lysates were centrifuged at 15,000 × *g* at 4 °C for 10 min, the supernatants were subjected to SDS-PAGE. After staining the gel with the Oriole^®^ fluorescent gel staining solution (1610496, Bio-Rad, Hercules, CA, USA), the major bands were cut on a UV transilluminator (Analytik Jena GmbH, Jena, Germany). The excised gels were sent to Japan Proteomics (Miyagi, Japan) for protein identification based on nano LC-MS/MS analysis.

### Plasmid construction and transfection

The pcDNA3-N-Flag-NLRP3 was purchased from Addgene (75127; Watertown, MA, USA). The DNA fragments encoding murine GSN, ASC, pro-caspase-1, and MAVS were obtained after PCR-based amplification of the cDNA from J774.1 cells. These fragments were subcloned into pcDNA3-6xMyc, pCS4-3xHA, or pcDNA3.1/V5-His A. Truncated mutant constructs of NLRP3 and GSN were generated by subcloning the PCR products from the plasmids expressing full-length NLRP3 and GSN into pcDNA3-N-Flag and pcDNA3-6xMyc, respectively. The constructs were transiently transfected into HEK293T cells for 48 h using Lipofectamine 2000 (11668027; Invitrogen) according to the manufacturer’s instructions. To chelate calcium, 5, 20, and 40 μM of BAPTA-AM (Calbiochem, San Diego, CA, USA) was added during the transfection.

### Co-immunoprecipitation (Co-IP)

To perform Co-IP of endogenous proteins, the BMDMs stimulated with LPS for 3 h were lysed in ice-cold cell lysis buffer (20 mM HEPES [pH 7.4], 150 mM NaCl, 1.5 mM MgCl_2_, 10 mM KCl, 1 mM EDTA, 1 mM EGTA, 1 mM DTT, and 0.5% Nonidet P-40) containing protease and phosphatase inhibitor cocktails (Nacalai). After the cell lysates were centrifuged at 15,000 × *g* at 4 °C for 10 min, the supernatants were incubated at 4 °C for 2 h with the anti-NLRP3 antibody (AG-20B-0014, AdipoGen, San Diego, CA, USA), which was conjugated with Dynabeads^®^ Protein G (Invitrogen). The beads were washed thrice with the lysis buffer. To perform Co-IP of the overexpressed proteins in HEK293T cells, the cells were transfected with the indicated plasmids as described earlier and lysed in ice-cold IP lysis buffer (50 mM HEPES [pH 7.0], 250 mM NaCl, 1 mM EDTA, and 0.2% Nonidet P-40) containing a protease inhibitor cocktail (Bio-Rad). The cell lysates were collected after centrifugation at 14,000 rpm for 15 min. The whole-cell lysates were subsequently incubated overnight with anti-Flag M2 affinity gel (Sigma) or anti-c-Myc agarose conjugate (Sigma) with rotation at 4 °C. The beads were extensively washed thrice with the IP lysis buffer. The immunoprecipitates were eluted with the IP elution buffer (0.1 M glycine HCl, pH 3.5) and neutralized with the IP neutralization buffer (0.5 M Tris HCl [pH 7.4] and 1.5 M NaCl). Both the whole cell lysates and immunoprecipitates were subjected to immunoblotting.

### In situ proximity ligation assay

The cells were seeded onto Lab-Tek^®^ II CC2^®^ eight-well chamber slides (154941PK; Thermo Fisher), stimulated with 100 ng/mL LPS for 3 h, fixed in 2% paraformaldehyde, and permeabilized 0.5% Triton X-100. Next, the cells were incubated for 17 h at 4 °C with the following antibodies: anti-GSN (12953, CST, Danvers, MA, USA), anti-NLRP3 (AG-20B-0014, AdipoGen), anti-MFN2 (9482, CST), and anti-MAVS (4983, CST). The cells were washed and allowed to react with a pair of Duolink proximity probes (Olink Bioscience, Uppsala, Sweden). The nuclei were counterstained with Hoechst 34580. Confocal images were acquired using a Zeiss LSM 980 laser scanning microscope (Carl Zeiss, Jena, Germany).

### Gel filtration chromatography

The BMDMs were primed with LPS with or without subsequent stimulation with nigericin. The cells were lysed in ice-cold NP-40 lysis buffer (50 mM Tris-HCl [pH 7.4], 1% NP-40, and 150 mM NaCl) containing a protease inhibitor cocktail (Bio-Rad) and centrifuged at 14,000 rpm for 15 min. Fresh soluble lysate containing 2.5 mg of total protein was loaded onto an XK 16/70 column packed with Superdex 200 prep-grade resin (Cytiva, Marlborough, MA, USA). The proteins were fractionated using an ÄKTA Pure Protein Purification System (Cytiva) in a buffer containing 20 mM sodium phosphate and 150 mM NaCl at pH 7.0. Each 5 mL fraction was collected and concentrated using an Amicon ultra centrifugal filter, and the concentrated samples were subjected to immunoblotting analysis.

### Immunoblotting analysis

Cells were lysed in RIPA buffer (CST) supplemented with a protease inhibitor cocktail (Bio-Rad). The whole cell lysates were collected through centrifugation at 14,000 rpm for 15 min. The proteins were quantified using the BCA assay (Pierce, Waltham, MA, USA). The proteins were separated using sodium dodecyl sulfate-polyacrylamide gel electrophoresis, and equal quantities of each protein were transferred onto nitrocellulose membranes. The membranes were probed with specific primary antibodies, followed by incubation with the corresponding secondary HRP-conjugated antibodies. The proteins were visualized using an ECL detection reagent (GE HealthCare, Chicago, IL, USA) and a luminescent image analyzer (ImageQuant LAS 4000). For protein precipitation from the cell culture supernatant, equal volumes of methanol and one-fourth volume of chloroform were added to the supernatant, followed by thorough mixing by vortexing and centrifugation at 13,000 rpm for 5 min. The upper aqueous phase was discarded, and an equal volume of methanol was added to each sample. After centrifugation at 13,000 rpm for 5 min, the protein pellet was dried at 55 °C and boiled in 1× SDS buffer containing 0.1 M DTT. Mouse plasma samples were boiled in 1× SDS buffer, and 5 μL of each plasma sample was equally loaded onto sodium dodecyl sulfate-polyacrylamide gel. The following antibodies were used: anti-NLRP3 (AG-20B-0014, AdipoGen), anti-GSN (12953, CST), anti-pGSN (ab75832, Abcam, Cambridge, UK), anti-ASC (AG-25B-0006, AdipoGen), anti-IL-1β (AF-401-NA, R&D systems, Minneapolis, MN, USA), anti-caspase-1 (AG-20B-0042, AdipoGen), caspase-11 (ab180673, Abcam, Cambridge, UK), anti-β-actin (4967, CST), anti-GAPDH (sc-25778, Santa Cruz, Dallas, TX, USA), anti-acetylated-α-tubulin (sc-23950, Santa Cruz), anti-p65 (8242, CST), anti-IκBα (ab32518, Abcam), anti-phospho-IκBα (9246, CST), anti-GSDMD (ab209845, Abcam), anti-TOMM20 (ab56783, Abcam), anti-α-tubulin (E12-054, EnoGene, New York, NY, USA), anti-TSG101 (ab125011, Abcam), anti-Flag (F7425, Sigma), anti-Flag (F1804, Sigma), anti-Myc (C3956, Sigma), anti-HA (11867423001, Roche, Basel, Switzerland), and anti-His (ab9108, Abcam). All primary antibodies were diluted in a 1:1000 ratio.

### Elisa

The cytokines concentrations in the cell culture supernatants, peritoneal lavage fluid, and plasma were measured using ELISA kits for mouse IL-1β (BioLegend, San Diego, CA, USA), mouse IL-18 (Invitrogen), mouse tumor necrosis factor alpha (TNFα) (BioLegend), and mouse GSN (ABclonal, Woburn, MA, USA), according to the manufacturer’s instructions.

### RNA isolation and quantitative real-time PCR

Total RNA was extracted using the TRIzol reagent (Invitrogen), and cDNA was synthesized by reverse transcription of 2 μg of RNA. Quantitative real-time PCR was performed using SYBR Green (Invitrogen) and detected on a LightCycler480 II (LC480; Roche) under the following cycling conditions: pre-incubation at 95 °C for 5 min, followed by 45 cycles of amplification at 95 °C for 10 s, 60 °C for 10 s, and 72 °C for 10 s. *Gapdh* was used as the internal control, and the relative gene expression was calculated using the 2^−∆∆Ct^ method. Specific primer sequences used for qRT-PCR are listed in the supplementary Table S1.

### Cytotoxicity assay

An LDH cytotoxicity assay kit (DoGenBio, Seoul, Korea) was used according to the manufacturer’s instructions to determine the release of lactate dehydrogenase (LDH) into the culture medium after NLRP3 inflammasome activation. For real-time cell death analysis, the LPS-primed cells were seeded into a 96-well plate at a density of 0.5 × 10^5^ cells/well. After stimulation with 5 μM nigericin and staining with Cytotox Green, the cells were imaged every 20 min for 3 h using an IncuCyte Live-Cell Analysis System (Sartorius, Göttingen, Germany).

### ASC oligomerization assay

Cells were lysed with PBS containing 0.5% Triton X-100 and a protease inhibitor cocktail (Bio-Rad), followed by centrifugation at 6000 × *g* for 15 min. The Triton X-100 soluble supernatant was collected, whereas the Triton X-100 insoluble pellet was washed twice with PBS and cross-linked at 37 °C for 30 min in PBS containing 2 mM disuccinimidyl suberate (Thermo Fisher Scientific) and a protease inhibitor cocktail (Bio-Rad). The cross-linked pellet was dissolved in 1× SDS buffer and boiled. The Triton X-100 soluble and insoluble fractions were subjected to immunoblotting analysis.

### Reconstitution of the NLRP3 inflammasome system in HEK293T cells

HEK293T cells were plated into 6-well plates at a density of 1.8 × 10^6^ cells/well and transfected with plasmids expressing Myc-GSN (500 ng), HA-ASC (350 ng), Flag-NLRP3 (350 ng), and Pro-caspase-1-V5 (1 μg) using Lipofectamine 2000 (11668027, Invitrogen). After 48 h, the cells were stimulated with nigericin (20 μM for 45 min), and cleaved pro-caspase-1 was assessed using immunoblotting analysis.

### Subcellular fractionation

Cytosolic and mitochondrial fractions were obtained for cultured cells (Thermo Fisher Scientific) using a mitochondrial isolation kit by following the manufacturer’s protocol. Each fraction was analyzed using immunoblotting and verified using anti-TOMM20 and anti-α-tubulin antibodies.

### Immunofluorescence cell staining

Cells were seeded onto Lab-Tek^®^ II CC2^®^ eight-well chamber slides (Thermo Fisher Scientific) and stimulated with 100 ng/mL LPS for 3 h. For some experiments, the LPS-primed macrophages were stimulated with nigericin (5 μM) for 15 min. Then, the cells were fixed in 2% paraformaldehyde and permeabilized in 0.5% Triton X-100. Subsequently, the cells were incubated for 17 h at 4 °C with the following primary antibodies: anti-NLRP3 (AG-20B-0014, AdipoGen), anti-MAVS (4983, CST), anti-ASC (67824), and anti-GRP78 (11587-1-AP, Proteintech). Next, the cells were incubated for 40 min at room temperature with the following secondary antibodies: Alexa Fluor 488-conjugated anti-rabbit IgG pAb (A11070; Invitrogen) and Alexa Fluor 546-conjugated anti-mouse IgG pAb (A11018, Invitrogen). For some experiments, the mitochondria were labeled with MitoSpy^®^ Orange CMTMRos (424803, BioLegend) and the nuclei were counterstained with Hoechst 34580. Confocal images were acquired using a Zeiss LSM 980 laser scanning microscope (Carl Zeiss) and analyzed using the Zen software (Carl Zeiss).

### Quantification of intracellular calcium

For intracellular calcium analysis using a microplate reader, LPS-primed peritoneal-resident macrophages were plated into 96-well black plates with a clear bottom at a density of 1 × 10^5^ cells/well. The cells were stained with 1 μM Fluo-4 AM (Invitrogen) for 30 min. The cells were stimulated with 20 μM nigericin, 1 mM CaCl_2_, and 5 μM ionomycin at the indicated times. Fluorescence was read for 30 min at 15 s intervals using the FLUOstar Omega microplate reader at 485 nm excitation and 520 nm emission wavelengths. For intracellular calcium analysis using confocal microscopy, LPS-primed peritoneal resident macrophages were plated onto 8-well chambered coverslips (Thermo Fisher Scientific) at a density of 1 × 10^6^ cells/well and stained with 5 μM Fluo-4 AM (Invitrogen) for 30 min. Images of untreated cells were acquired (*t* = 0). Subsequently, the cells were stimulated with 20 μM nigericin or 1 mM CaCl_2_ and imaged for 30 min at 15 s intervals. After 30 min, 5 μM ionomycin was added to the cells, and the cells were imaged for 10 min at 15 s intervals. Fluorescence images were obtained using a Zeiss LSM 980 laser scanning microscope (Carl Zeiss) with a 488 nm laser and emission at 525 nm. The images were analyzed using the Imaris software (Andor Technology, Belfast, Northern Ireland).

### Mitochondrial function assays

To measure mitochondrial damage, the cells were co-stained with 200 nM MitoTracker Green (Invitrogen) and 200 nM MitoTracker Deep Red (Invitrogen) for 30 min. After washing twice with PBS, the cells were resuspended in PBS and subjected to flow cytometric analysis using an LSRFortessa X-20 flow cytometer (BD Biosciences, Franklin Lakes, NJ, USA). To measure mitochondrial ROS production, the cells were stained with 5 μM MitoSOX Red (Invitrogen) for 15 min. After washing twice with PBS, the cells were resuspended in PBS and subjected to flow cytometric analysis using an LSRFortessa X-20 flow cytometer (BD Biosciences).

### Transmission electron microscopy (TEM)

Specimens were fixed in a solution containing 2% glutaraldehyde and 2% paraformaldehyde in 0.1 M phosphate buffer (pH 7.4) for 12 h. Subsequently, the specimens were washed in 0.1 M phosphate buffer, post-fixed with 1% OsO_4_ in 0.1 M phosphate buffer for 2 h, and dehydrated with an ascending series of ethanol (50%, 60%, 70%, 80%, 90%, 95%, and 100%) for 10 min each. Next, the specimens were incubated with propylene oxide for 10 min, embedded with a Poly/Bed 812 kit (Polysciences, Warrington, PA, USA), and polymerized in an electron microscope oven (TD-700, Dosaka, Kyoto, Japan) at 65 °C for 12 h. The resulting block was sliced (200-nm semi-thin sections) with a diamond knife in an ultramicrotome and stained with toluidine blue for observation under an optical microscope. The region of interest was subsequently cut into 80-nm thin sections using an ultramicrotome, placed on copper grids, double-stained with 5% uranyl acetate for 20 min and 3% lead citrate for 7 min, and imaged using a transmission electron microscope (HT7800; Hitachi, Tokyo, Japan) equipped with an RC10 CMOS camera at an acceleration voltage of 80 kV.

### Flow cytometry

The PECs were stained with PerCP-Cyanine5.5-anti-CD11b Invitrogen BV421-anti-CD11b (BioLegend), BV786-anti-CD11c (BioLegend), BV605-anti-CD19 (BioLegend), FITC-anti-Ly6C (BD Biosciences), APC-anti-Ly6G (Invitrogen), YG-PE-anti-F4/80 (BioLegend), and 7-AAD (BioLegend), followed by flow cytometry using an LSRFortessa X-20 (BD Biosciences) instrument. The data were analyzed using the FlowJo software (BD Biosciences).

### Exosome isolation

To purify the exosomes, BMDMs were cultured in media supplemented with 10% exosome-free FBS and ultracentrifuged at 100,000 × *g* for 24 h. After priming with LPS and stimulation with nigericin, the exosomes were isolated from the cell culture supernatant using Total Exosome Isolation Reagent (4478359, Thermo Fisher Scientific) by following the manufacturer’s instructions. Then, the exosome samples were subjected to immunoblotting analysis.

### Adjuvant-induced arthritis

Male mice aged 6–10 weeks were intradermally injected on day 0 with 100 µL of either complete Freund’s adjuvant (2 mg/mL of heat-killed *Mycobacterium tuberculosis*, Chondrex, Woodinville, WA, USA) or incomplete Freund’s adjuvant (control, Chondrex). Arthritic severity and the diameters of the ankle and sole to instep were measured and scored daily. Arthritic severity was graded from 0 to 4, as previously described (Moudgil, Diversification of T cells). On day 8, both paws and plasma samples were collected for hematoxylin and eosin (H&E) staining, immunoblotting analysis, and ELISA.

### MSU-induced peritonitis

Male mice aged 6–10 weeks were intraperitoneally injected with 1 mg of either MSU crystals (Invivogen) dissolved in sterile PBS or sterile PBS as the control. After 6 h, the peritoneal cavities were washed with 10 mL of sterile PBS, and the peritoneal lavage fluid was collected. The supernatants were concentrated using an Amicon ultra centrifugal filter, followed by the assessment of the cytokine levels using ELISA. The PECs were analyzed using fluorescence-activated cell sorting (FACS) to determine immune cell infiltration.

### Folic acid-induced acute tubular necrosis

Male mice aged 6–10 weeks were intraperitoneally injected with 250 mg/kg folic acid (Sigma) dissolved in 150 mM sodium bicarbonate. After 40 h, the mice were weighed and euthanized through CO_2_ asphyxiation. Blood samples were collected via cardiac puncture, and the kidneys were flushed with PBS prior to removal. Blood urea nitrogen (BUN) concentrations in serum samples were determined using a BUN colorimetric detection kit (K024-H1, Arbor Assays, Ann Arbor, MI, USA).

### LPS-induced septic shock

Male mice aged 6–10 weeks were intraperitoneally injected with 20 mg/kg LPS (*E. coli* O111:B4; Sigma) or the same volume of sterile PBS (control). After 3 h, the plasma and peritoneal lavage fluid were collected. The peritoneal lavage fluid was concentrated using an Amicon Ultra centrifugal filter, and both plasma and peritoneal lavage fluid samples were analyzed using ELISA. For the survival test, mice were intraperitoneally injected with 10 mg/kg of LPS (*E. coli* O111:B4; Sigma) or PBS (control). The mice were monitored every 6 h for 96 h.

### Histology

Sagittally transected kidneys were fixed with 4% paraformaldehyde in PBS at 4 °C for 16 h, followed by continuous immersion in 10% sucrose in PBS at 4 °C for 5 h and 20% sucrose in PBS at 4 °C for 16 h. The samples were embedded in OCT compound (Sakura Finetek, Torrance, CA, USA) and stored at −80 °C. The frozen sections (8-mm thick) were subjected to immunofluorescence staining and H&E staining. For immunofluorescence staining, the sections were incubated for 17 h at 4 °C with the following primary antibodies: anti-Ly-6G (127601, BioLegend) and anti-F4/80 (Ab00106-2.3, Absolute Antibody, Oxford, UK). Then, they were stained with Alexa Fluor 488-conjugated anti-rat IgG pAb (A11006, Invitrogen) and Alexa Fluor 546-conjugated anti-mouse IgG pAb (A11018, Invitrogen) for 40 min at room temperature (RT). The nuclei were counterstained with Hoechst 34580. Confocal images were acquired using a Zeiss LSM 980 laser scanning microscope (Carl Zeiss) and analyzed using Zen software (Carl Zeiss). The sections were stained with Mayer’s hematoxylin and eosin (Sakura Finetek).

### Analysis of nanopore long read sequencing data

The nanopore reads were retrieved and explored via the UCSC Genome Browser platform. The expression ratio of cGSN and pGSN transcripts was calculated by referencing the gene annotations provided by GENCODE Release M34. The analysis focused on distinguishing cGSN and pGSN the transcripts based on their annotated genomic coordinates and using the isoform-specific sequence features.

### Quantification and Statistical Analysis

All data were analyzed using GraphPad Prism 5 (GraphPad Software, Inc., La Jolla, CA, USA). The data are presented as mean ± SD or mean ± SEM. Statistical significance was evaluated using a two-tailed Student’s *t* test or Two-way ANOVA followed by additional validation using Bonferroni method. For the survival analysis, statistical significance was assessed using the log-rank test. *P* < 0.05 were considered significant.

## Supplementary information

Supplemental Information

Uncut Images for Figures

## Data Availability

Any information required to reanalyze the data reported in this study is available from the corresponding authors (LKKIM@yuhs.ac) upon request.
