# Supplementary material for: Gelsolin alleviates rheumatoid arthritis by negatively regulating NLRP3 inflammasome activation
Source: Cell Death Differ. 2024 Aug 24;31(12):1679–94. doi: 10.1038/s41418-024-01367-6 (PMC11618363; doi:10.1038/s41418-024-01367-6)
Supplement: Supplementary file 1 — Supplemental Information [file 41418_2024_1367_MOESM1_ESM.docx]

**Gelsolin Alleviates Rheumatoid Arthritis by Negatively Regulating NLRP3 Inflammasome Activation**

**Supplemental information**

Document S1. Figures S1–S9 and Table S1.

**
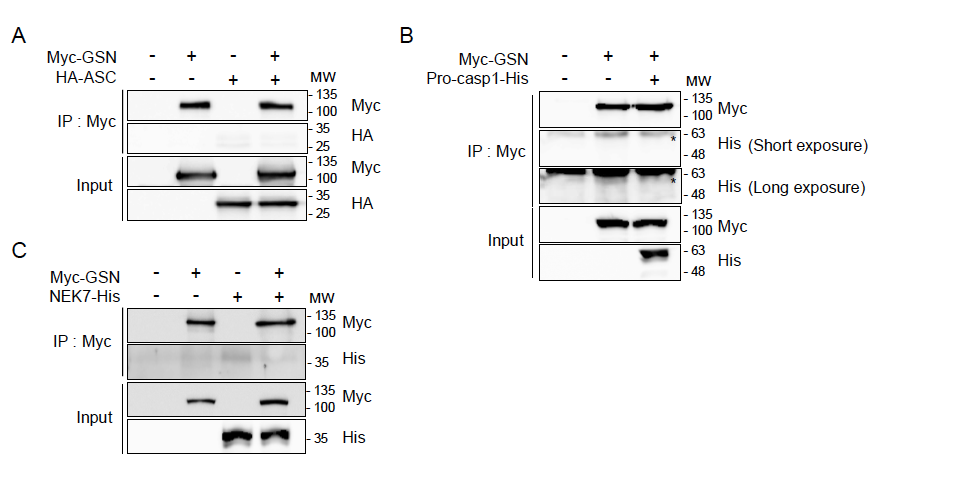
**

**Figure S1.** Association between gelsolin (GSN) and NLRP3 inflammasome components

Immunoblots of co-immunoprecipitated proteins with anti-Myc antibody in HEK293T cells that were transiently transfected with Myc-GSN, HA-ASC (A), Pro-casp1-His (B), and NEK7-His (C). Asterisks (*) indicate the heavy chain. Data shown in A–C are representative of at least three independent experiments.


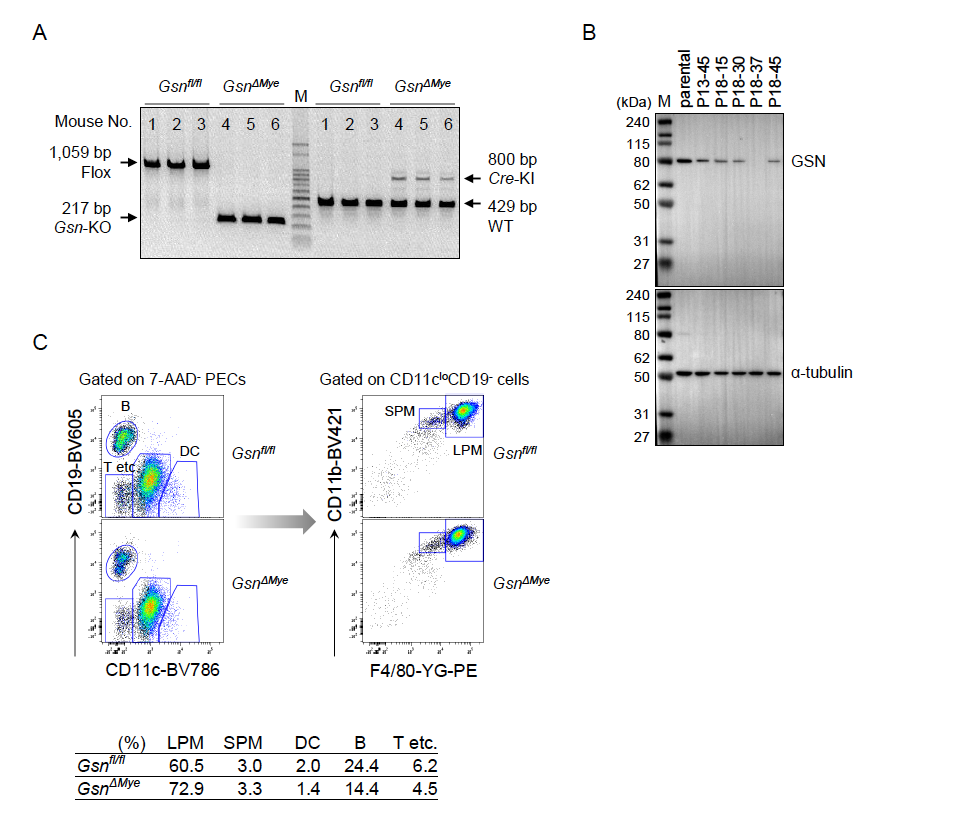


**Figure S2.** Generation of *Gsn*-KO J774.1 cells and *Gsn^ΔMye^* mice

(A) PCR analysis of DNA extracted from the mouse tail. PCR was performed using each primer set for *Gsn^fl/fl^* (left) and *Cre* (right). M, DNA size marker. (B) Immunoblots of whole cell lysates of parental J774.1 cells and cell clones transduced with *Gsn*-specific sgRNAs and Cas9 protein. M, protein molecular weight marker. (C) PECs collected from naïve mice were subjected to FACS analysis. B, B cells; DC, dendritic cells; T, T cells; SPM, small peritoneal macrophages; LPM, large peritoneal macrophages. Numerical values in the table indicate the percentage of 7-AAD-genative PECs.


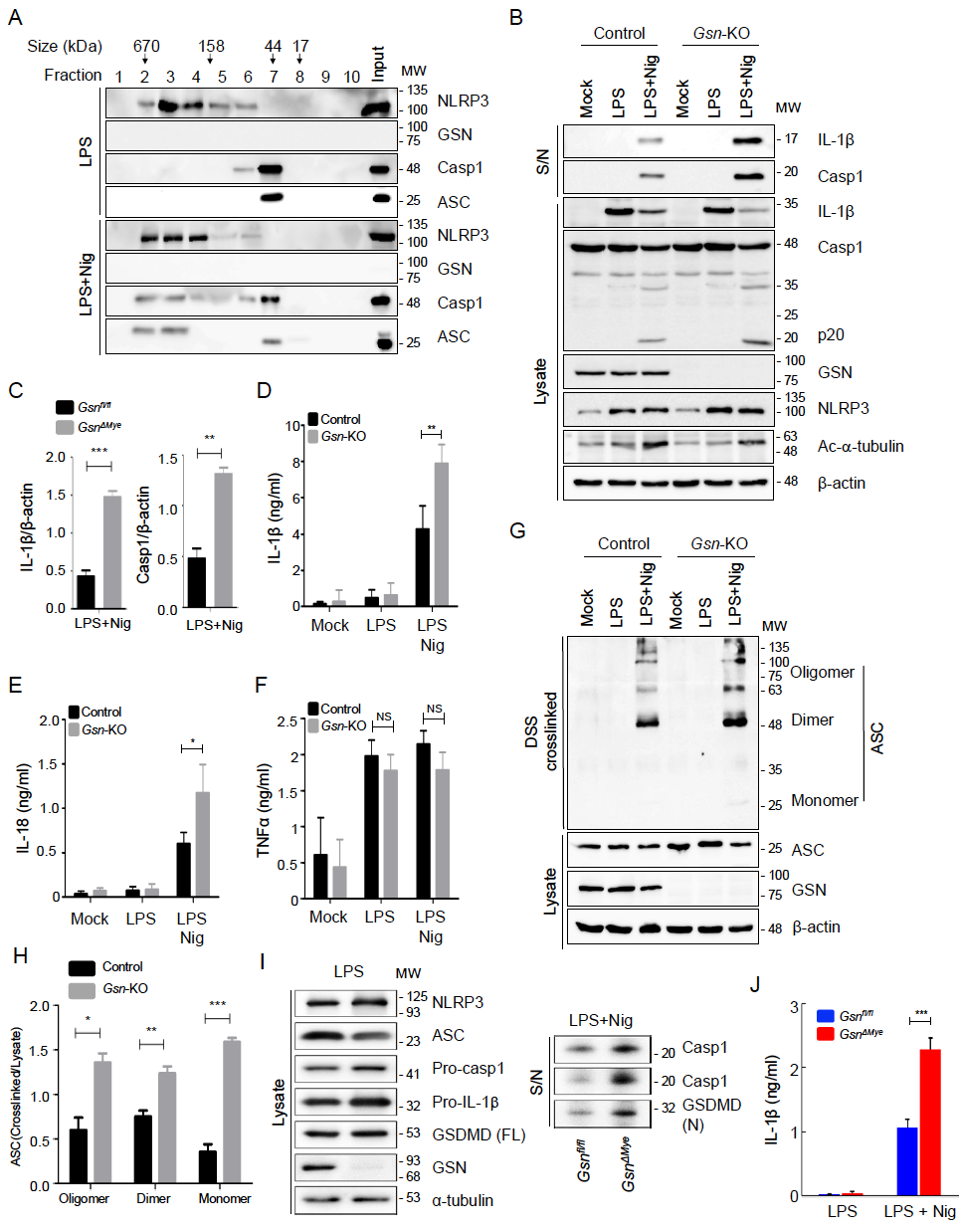


**Figure S3.** Loss of GSN leads to excessive NLRP3 inflammasome activation following nigericin stimulation

(A) Immunoblots of cell lysate of *Gsn^ΔMye^* BMDMs only primed with LPS (500 ng/mL, 3 h) and both primed and stimulated with nigericin (5 μM, 45 min), followed by fractionation using gel-filtration chromatography. (B,C) Immunoblots (B) and quantification (C) of supernatant (S/N) and cell lysates (Lysate) of control and *Gsn*-KO J774.1 cells left untreated, primed with LPS (500 ng/mL, 3 h), and primed and stimulated with nigericin (5 μM, 45 min). (D–F) ELISA of IL-1β (D), IL-18 (E), and TNFα (F) in the supernatant of control and *Gsn*-KO J774.1 cells left untreated, primed with LPS (500 ng/mL, 3 h), and primed and stimulated with nigericin (5 μM, 45 min) (n = 4). NS, not significant. (G, H) Immunoblots (G) and quantification (H) of ASC oligomerization of DSS cross-linked pellets and lysates in control and *Gsn*-KO J774.1 cells left untreated, primed with LPS (500 ng/mL, 3 h), and primed and stimulated with nigericin (5 μM, 45 min). (I) Immunoblots of cell lysates (Lysate) and supernatant (S/N) of *Gsn^fl/fl^* and *Gsn^ΔMye^* peritoneal resident macrophages only primed with LPS (100 ng/mL, 3 h) (upper) and both primed and stimulated with nigericin (5 μM, 2 h) (lower). (J) ELISA of IL-1β in the culture supernatants of *Gsn^fl/fl^* and *Gsn^ΔMye^* peritoneal resident macrophages only primed with LPS (100 ng/mL, 3 h) and both primed and stimulated with nigericin (5 μM, 2 h) (n = 3). Data are presented as mean ± SD (C–F, H, J). Student’s *t*-test, **P* < 0.05, ***P* < 0.01, ****P* < 0.001 (C-F, H, J). Data shown in B, G, and I are representative of at least three independent experiments.


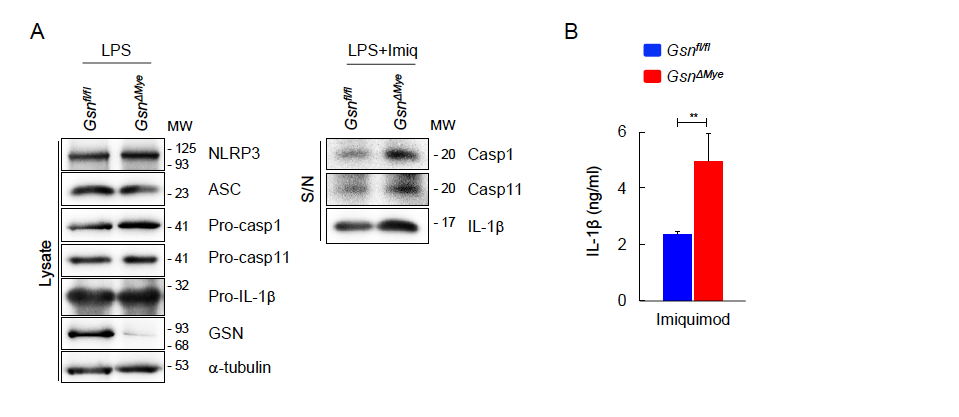


**Figure S4.** GSN deficiency leads to excessive NLRP3 inflammasome activation following imiquimod stimulation

(A) Immunoblots of cell lysates (Lysate) and supernatant (S/N) of *Gsn^fl/fl^* and *Gsn^ΔMye^* peritoneal resident macrophages only primed with LPS (100 ng/mL, 3 h) (left) and both primed and stimulated with imiquimod (5 µg/mL, 2 h) (right). (B) ELISA of IL-1β in the culture supernatants of *Gsn^fl/fl^* and *Gsn^ΔMye^* peritoneal resident macrophages primed with LPS (100 ng/mL, 3 h) and stimulated with imiquimod (5 µg/mL, 2 h). Data are presented as mean ± SD of three independent experiments (B). Student’s *t*-test, ***P* < 0.01 (B).


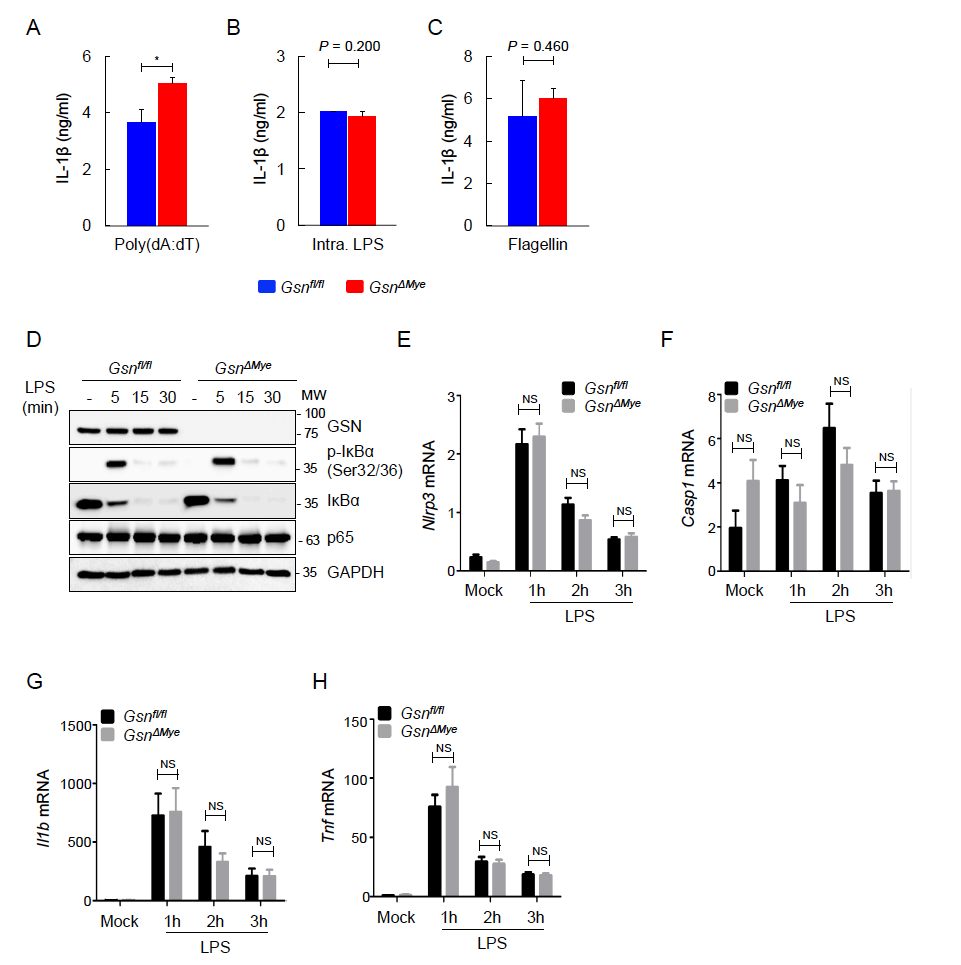


**Figure S5.** GSN does not affect the non-canonical and NLRC4 inflammasomes or the NF-κB pathway

(A–C) ELISA of IL-1β in the culture supernatants of Gsnfl/fl and GsnΔMye peritoneal resident macrophages primed with LPS (100 ng/mL, 3 h) and stimulated with poly(dA:dT) (0.75 µg/mL, 2 h) (A), LPS (2 µg/mL, 2 h) (B), or flagellin (1.5 µg/mL, 2 h) (C). (D) Immunoblots for IκBα, the phosphorylated form of the serine 32 and 36 region of IκBα, and p65 in cell lysates of *Gsn^fl/fl^* and *Gsn^ΔMye^* BMDMs left untreated or primed with LPS (500 ng/mL) for the indicated time. (E–H) qRT-PCR analysis for mRNA expression of the indicated genes. mRNA expression of each gene was normalized to the internal control *Gapdh* (n = 19). NS, not significant. Data are presented as mean ± SD of three independent experiments (A-C) or mean ± SEM (E–H). Student’s *t*-test, **P* < 0.05 (A). Data shown in D are representative of at least three independent experiments.


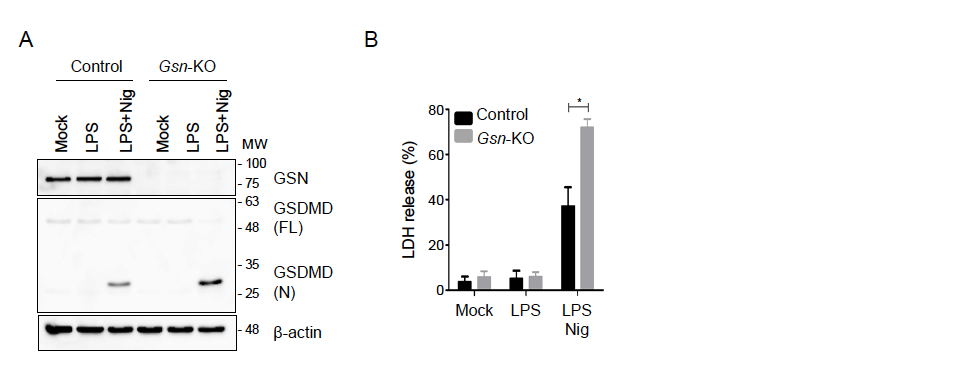


**Figure S6.** Loss of GSN promotes pyroptotic cell death

(A) Immunoblots of full length and cleaved GSDMD in cell lysates of control and *Gsn*-KO J774.1 cells left untreated, primed with LPS (500 ng/mL, 3 h), and primed and stimulated with nigericin (5 μM, 45 min). (B) LDH released into the supernatant of control and *Gsn*-KO J774.1 cells left untreated, primed with LPS (500 ng/mL, 3 h), and primed and stimulated with nigericin (5 μM, 45 min) (n = 3). Data are presented as mean ± SEM (B). Student’s *t*-test, **P* < 0.05 (B). Data shown in A are representative of at least three independent experiments.


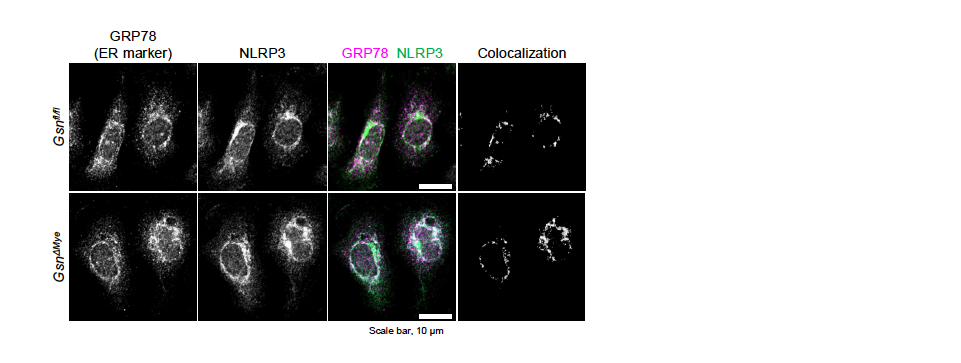


**Figure S7.** GSN depletion causes changes in the intracellular distribution of the ER

Immunofluorescence of co-staining of GRP78 and NLRP3 in *Gsn^fl/fl^* and *Gsn^ΔMye^* peritoneal resident macrophages primed with LPS (50 ng/mL, 3 h).


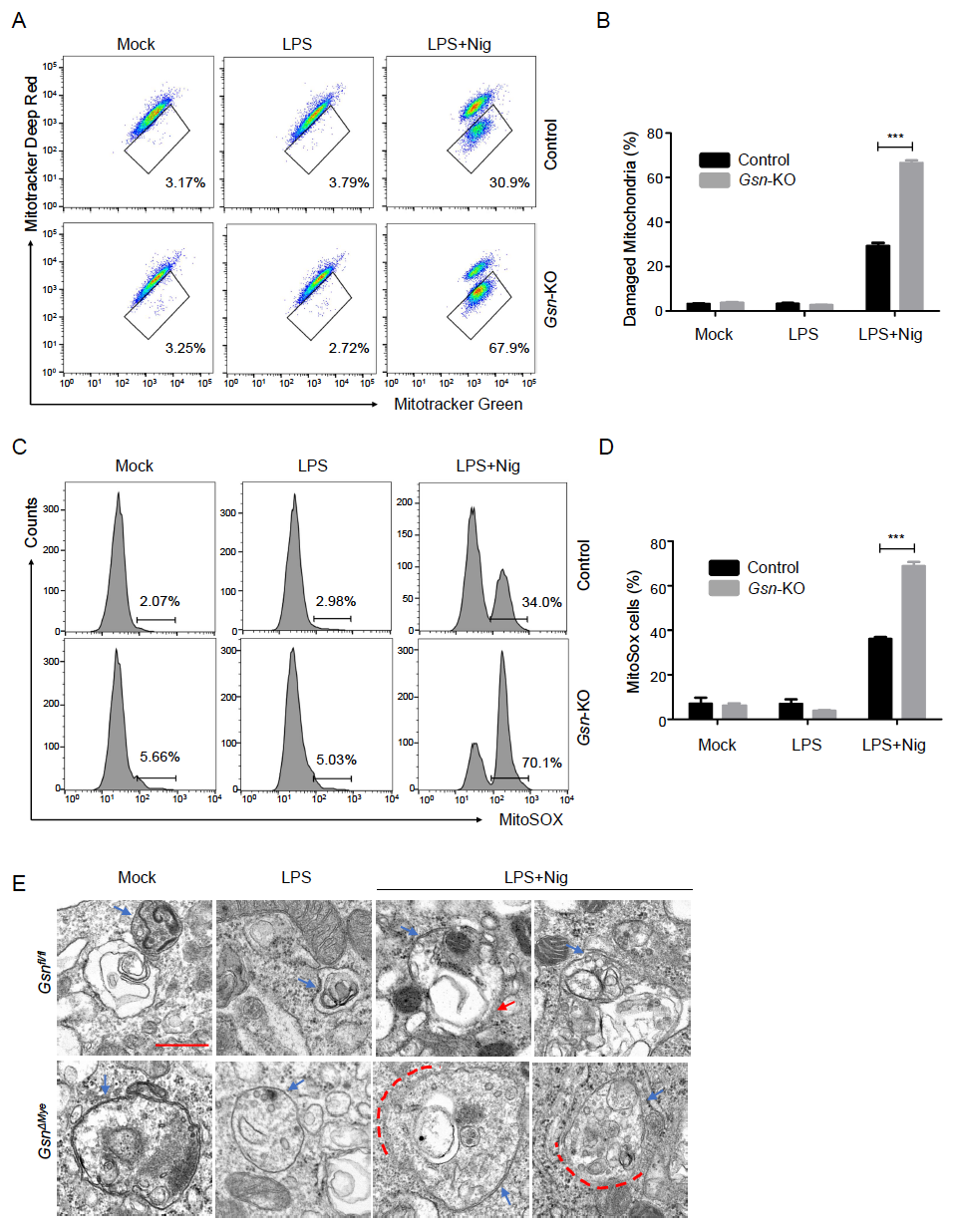


**Figure S8.** Loss of GSN promotes mitochondrial damage

Representative dot plots (A) and histograms (C) of flow cytometry and quantification (B, D) of damaged mitochondria (surrounded by black lines in A) (n = 4) and MitoSOX^+^ mitochondria (indicated by black bars in C) (n = 4) in untreated control and *Gsn*-KO J774.1 cells left untreated, primed with LPS (500 ng/mL, 3 h), and primed and stimulated with nigericin (5 μM, 45 min). Data are presented as mean ± SEM (B, D). (E) Representative TEM images of mitochondria from *Gsn^fl/fl^* and *Gsn^ΔMye^* peritoneal resident macrophages left untreated, primed with LPS (500 ng/mL, 3 h), and primed and stimulated with nigericin (5 μM, 45 min). To avoid bias, at least 10 images were acquired per sample. Blue arrows indicate lysosomal membranes. Red arrow and red dashed line designate areas that have no detectable membranes. The images were acquired at ×72.0 k magnification. Scale bar: 5 μm. Student’s *t*-test; ****P* < 0.001 (B, D). Data shown in A and C are representative of at least three independent experiments.


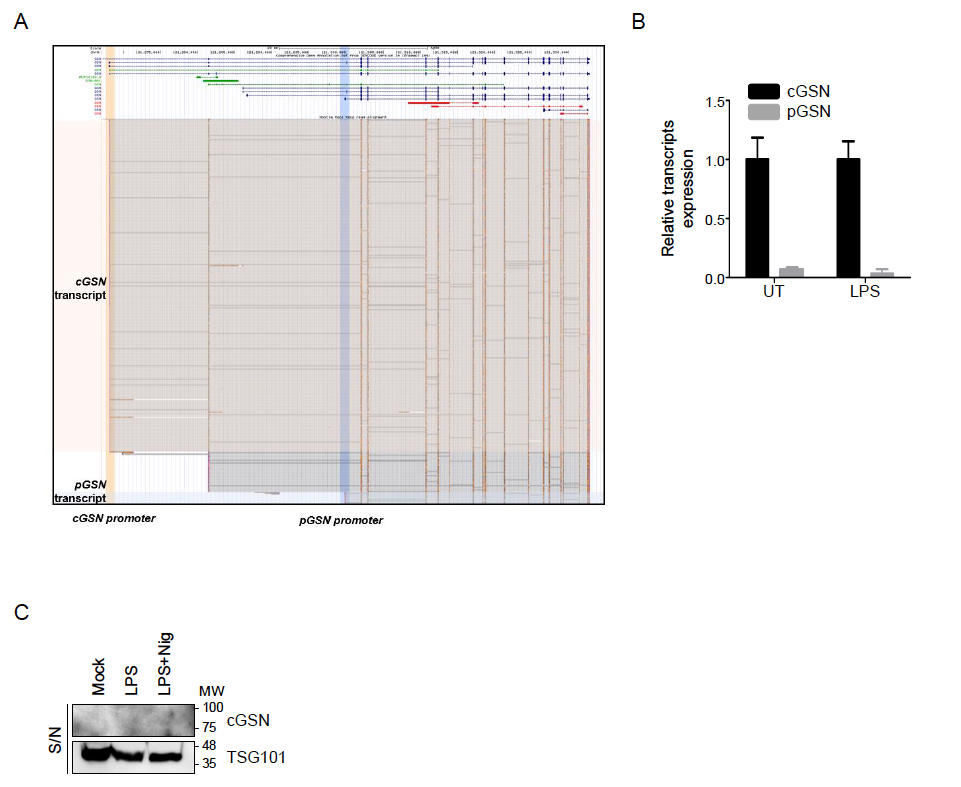


**Figure S9.** Nanopore sequencing data in GSN locus and exosome-dependent secretion of GSN

(A–B) Using the UCSC Genome Browser interface, nanopore sequencing reads for GSN in primary human monocyte-derived macrophages were acquired and analyzed. Gene annotations were based on the GENCODE version 34 (hg38) release. Representative images at steady state (A) and quantification of relative transcript expression for two replicates (B) are presented. In the graphical representation, a dark red box marks the location of the transcription start site for cGSN, whereas the transcription start site for pGSN is denoted by a dark blue box. Transcripts anticipated for cGSN and pGSN are encapsulated within boxes colored red and blue, respectively. (C) Immunoblots of isolated exosomes from the supernatant (S/N) of control J774.1 cells left untreated, primed with LPS (500 ng/mL, 3 h), and primed and stimulated with nigericin (5 μM, 45 min). Data are presented as means ± SEM (B).

**Table S1**

| Target |  | Sequences (5' → 3') |
| --- | --- | --- |
| *Gsn^fl/fl^* | forward | CTGAACTGAGTACTCTCTACACCAGGC |
|  | reverse | GTCAAATACTCATCACAGGGCCCTCG |
| *Cre* | forward (wild-typed) | GTCGGCCAGGCTGACTCCATAG |
|  | forward (*Cre*) | CCCAGAAATGCCAGATTACG |
|  | reverse (common) | GCATTGCAGACTAGCTAAAGGCAG |
| *Gapdh* | forward | GGCAAATTCAACGGCACAGTCAAG |
|  | reverse | TCGCTCCTGGAAGATGGTGATGG |
| *Nlrp3* | forward | GCCTACAGTTGGGTGAAATGT |
|  | reverse | GGAGGGCTTGATAGCAGTGA |
| *Casp1* | forward | TCCGCGGTTGAATCCTTTTCAGA |
|  | reverse | ACCACAATTGCTGTGTGT GCGCA |
| *Il1b* | forward | TCAACCAACAAGTGATATTCTC |
|  | reverse | ACACAGGAC AGGTATAGATTC |
| *Tnf* | forward | ATGTCCATTCCTGAGTTCTG |
|  | reverse | AATCTGGAAAGGTCTGAAGG |
