# Supplementary material for: Gelsolin alleviates rheumatoid arthritis by negatively regulating NLRP3 inflammasome activation
Source: Cell Death Differ. 2024 Aug 24;31(12):1679–94. doi: 10.1038/s41418-024-01367-6 (PMC11618363; doi:10.1038/s41418-024-01367-6)

Figure 1

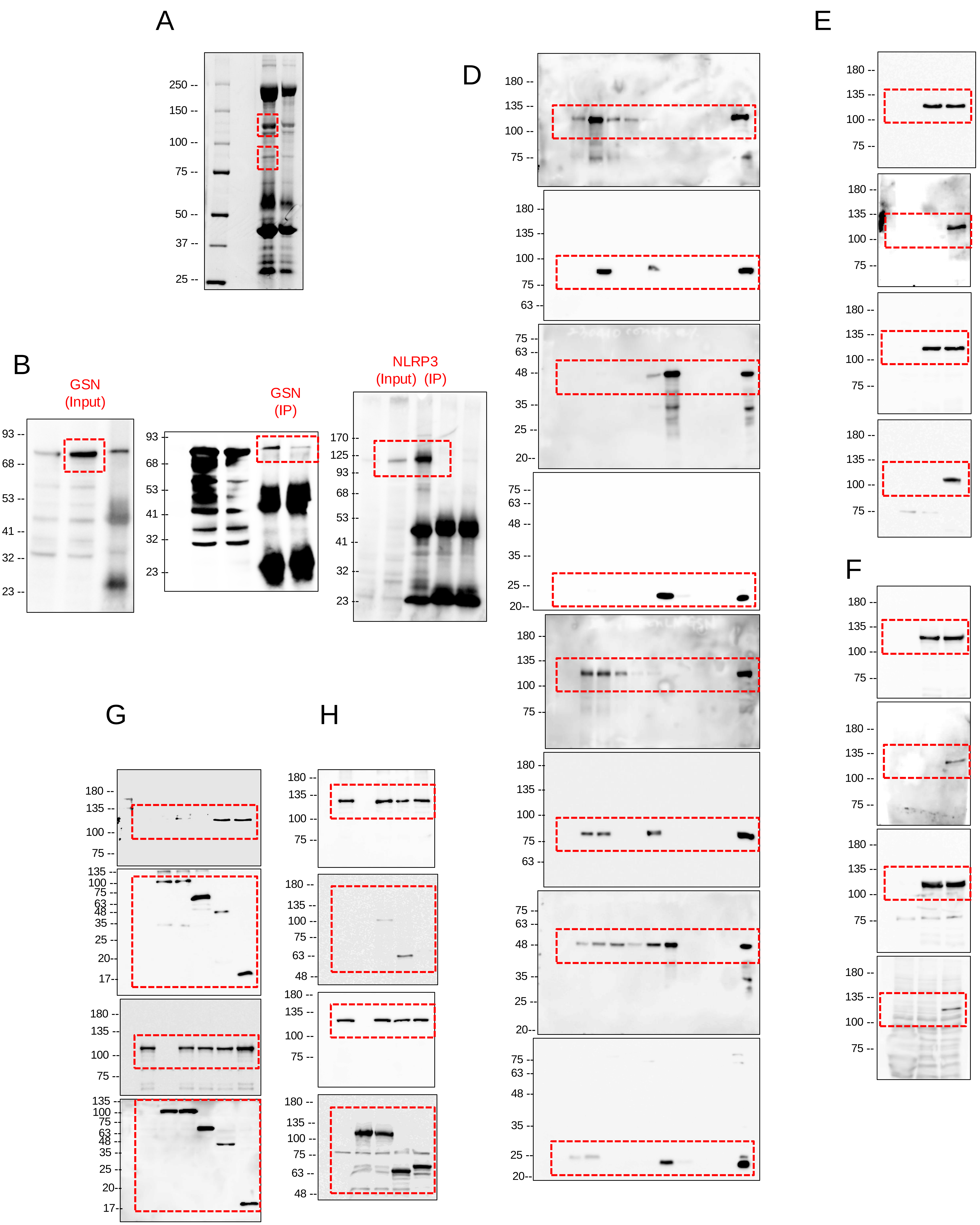

Figure 2

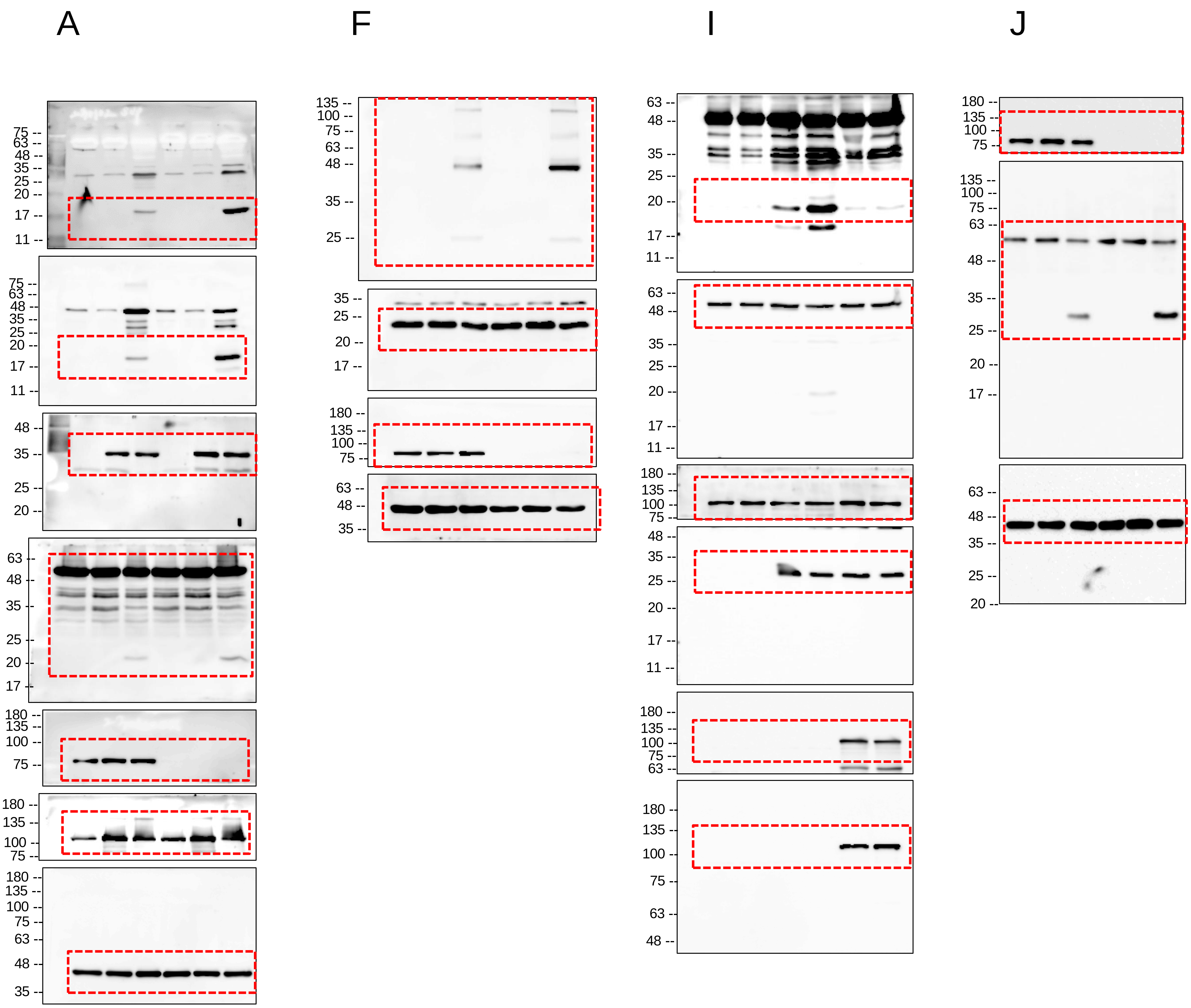

Figure 3

A

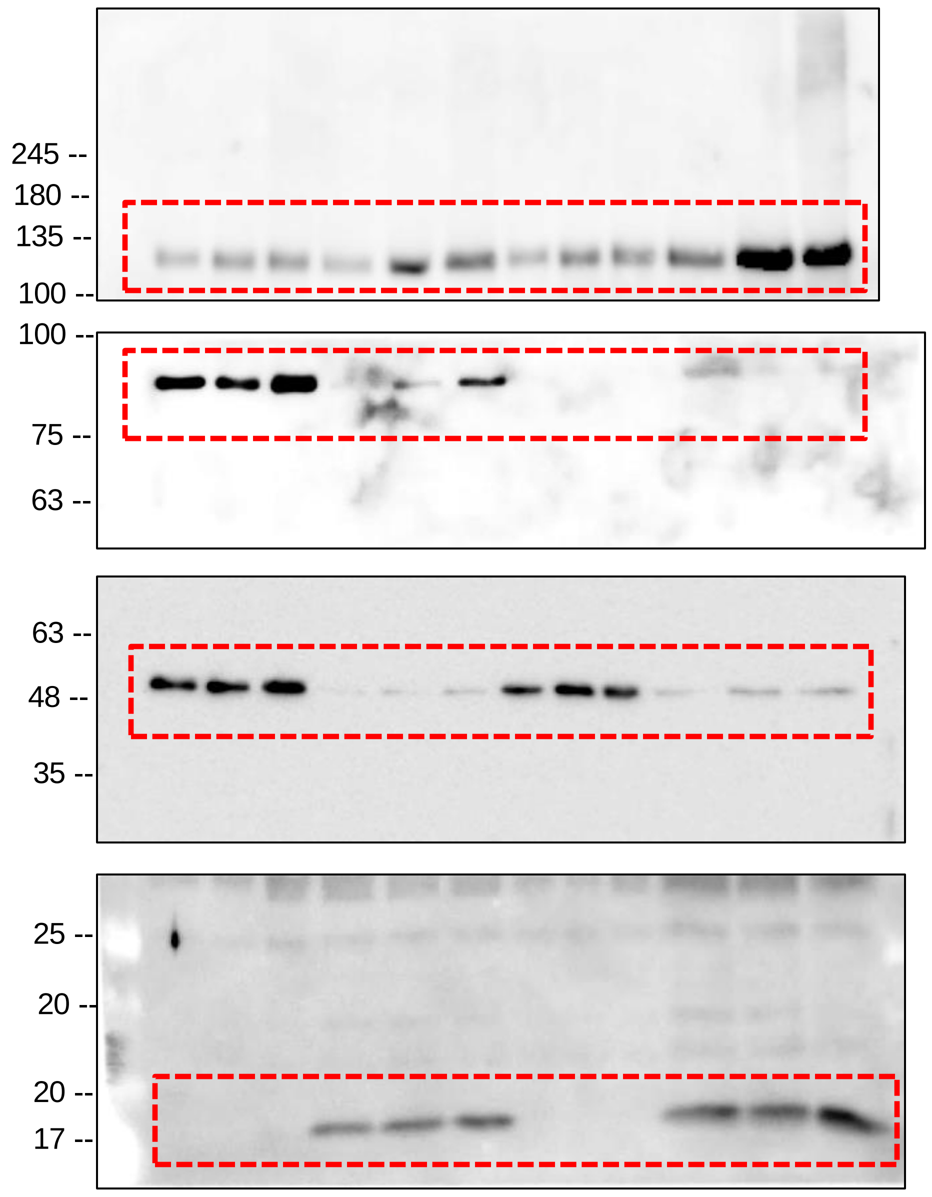

D

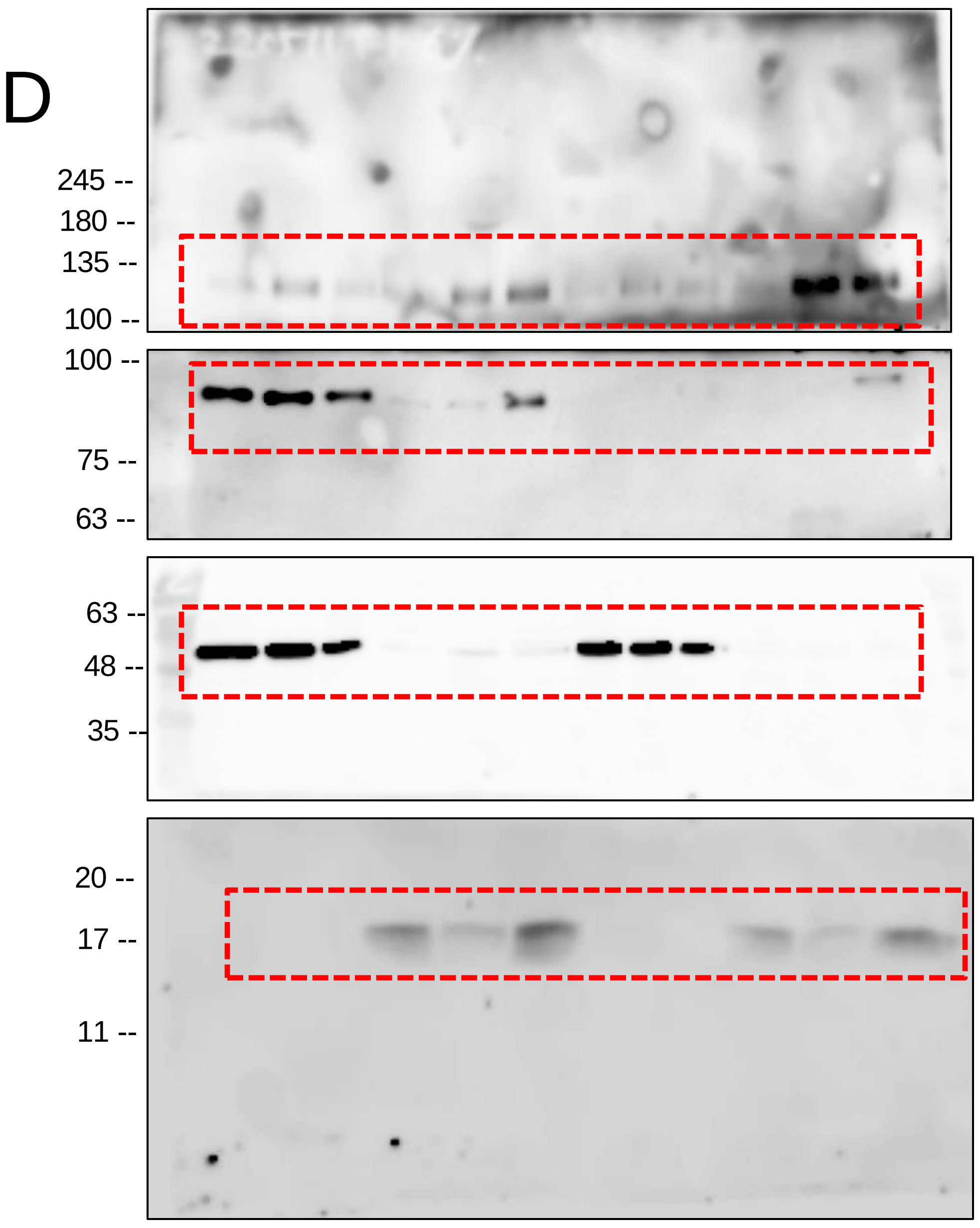

I

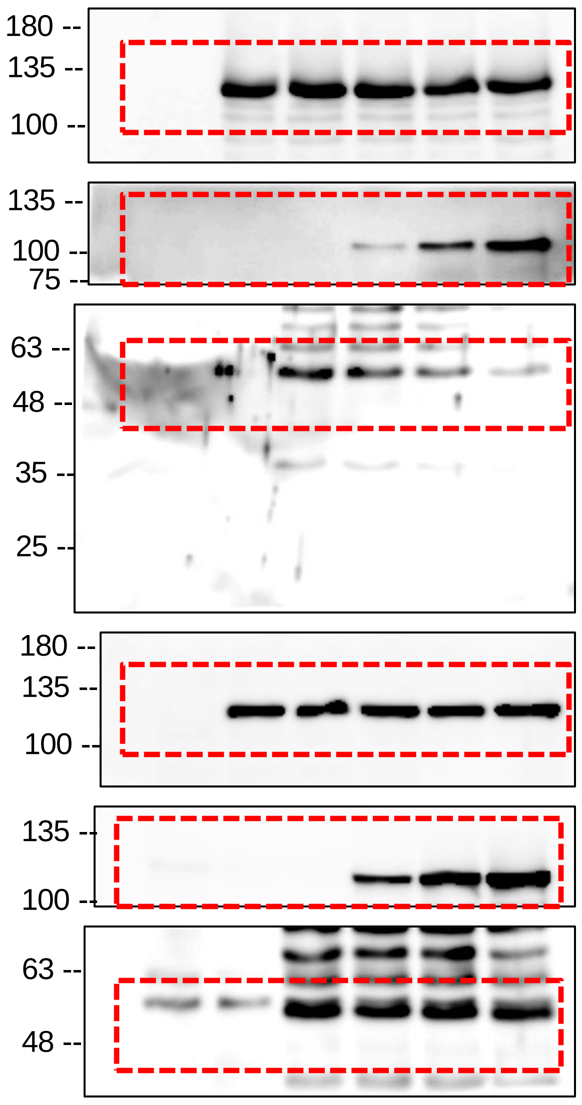

K

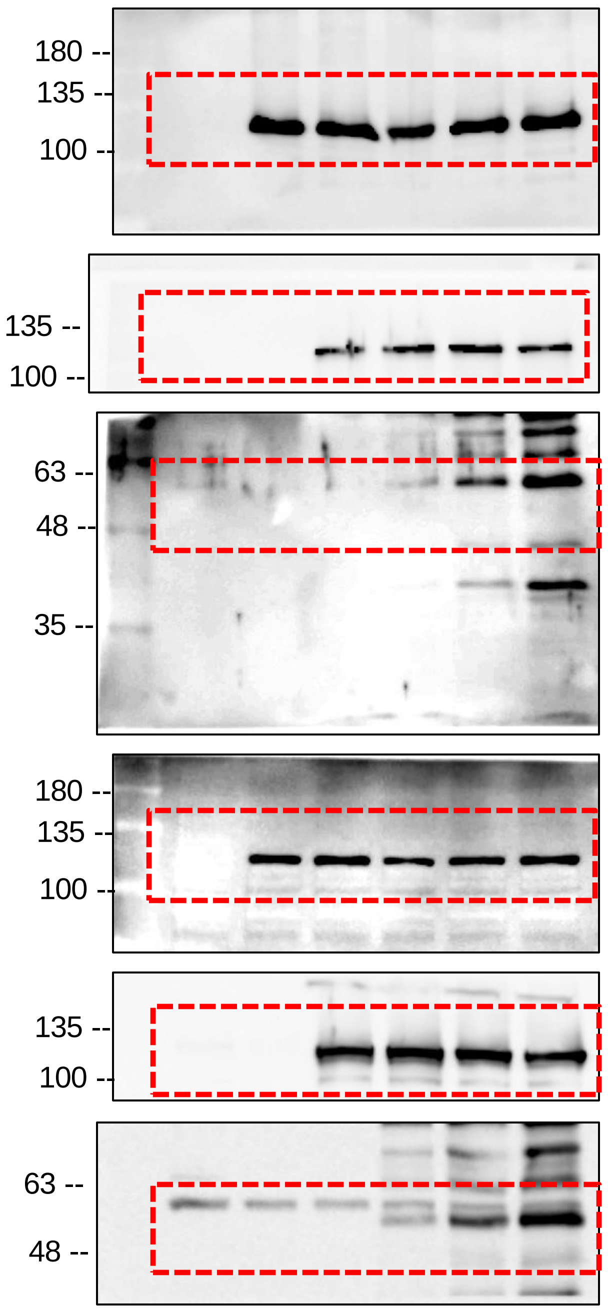

Figure 4

A

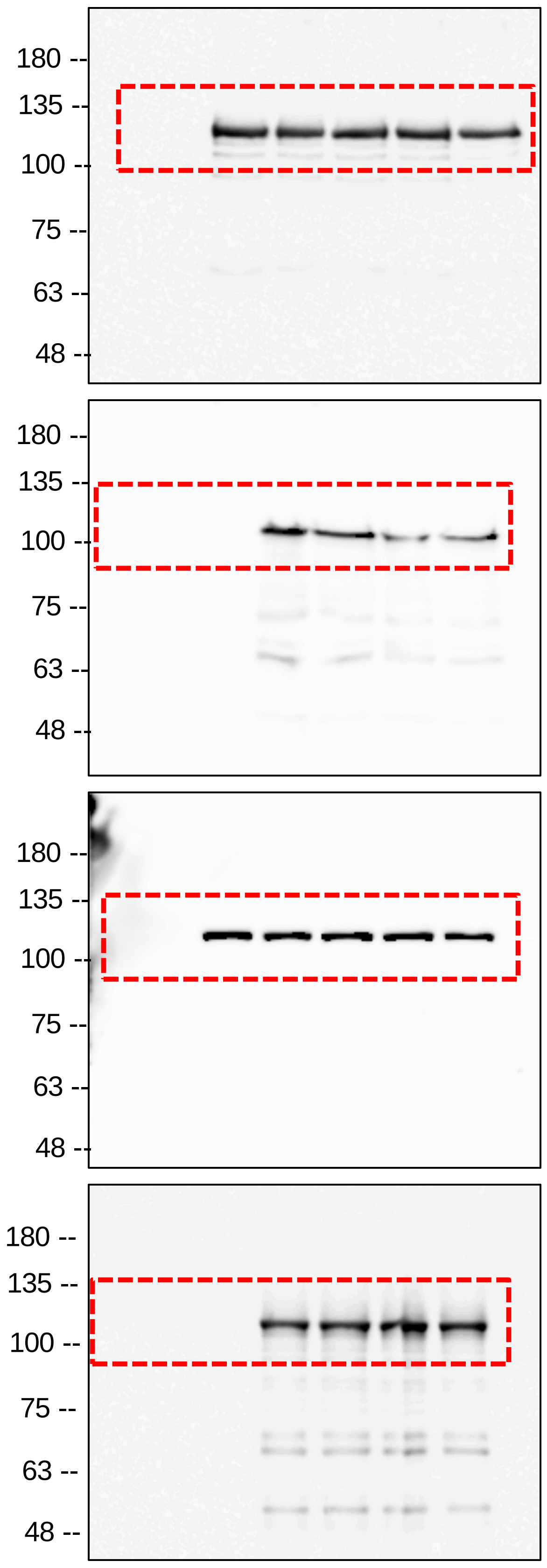

Figure 6

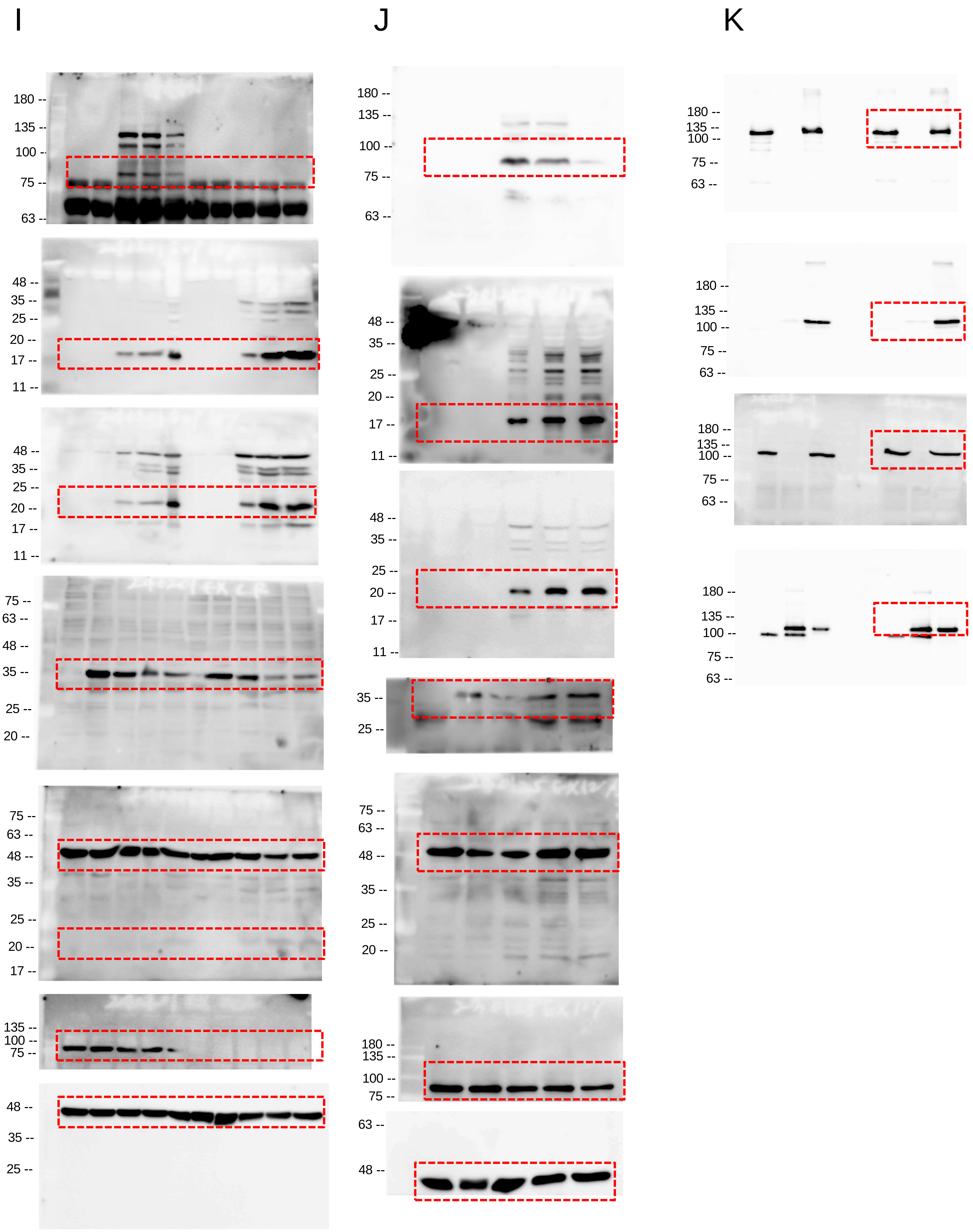

I
180 --
135 --
100 --
75 --
63 --
48 --
35 --
25 --
20 --
17 --
11 --
J
180 --
135 --
100 --
75 --
63 --
48 --
35 --
25 --
20 --
17 --
11 --
K
180 --
135 --
100 --
75 --
63 --

Supplementary Figure 1

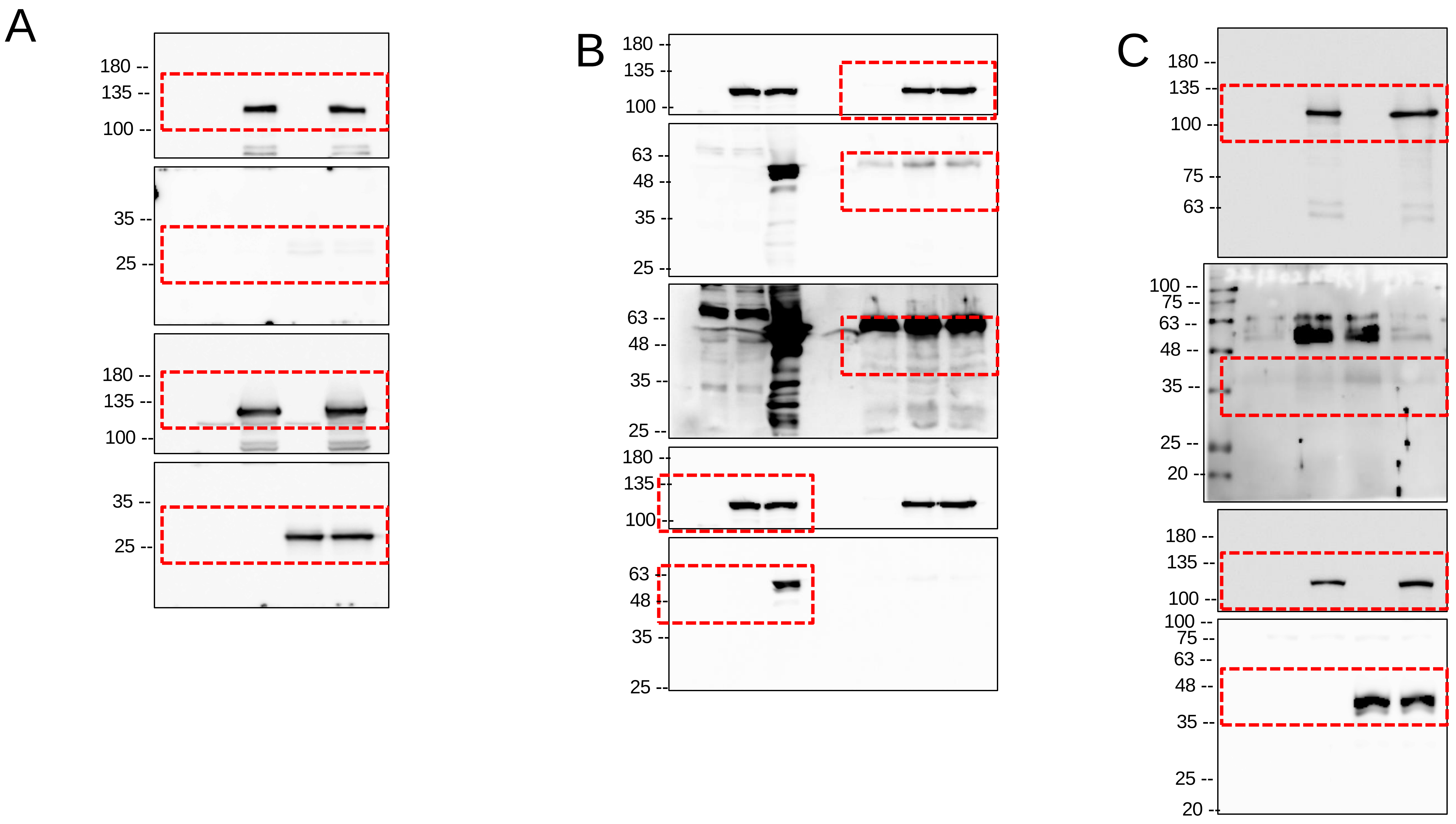

Supplementary Figure 3

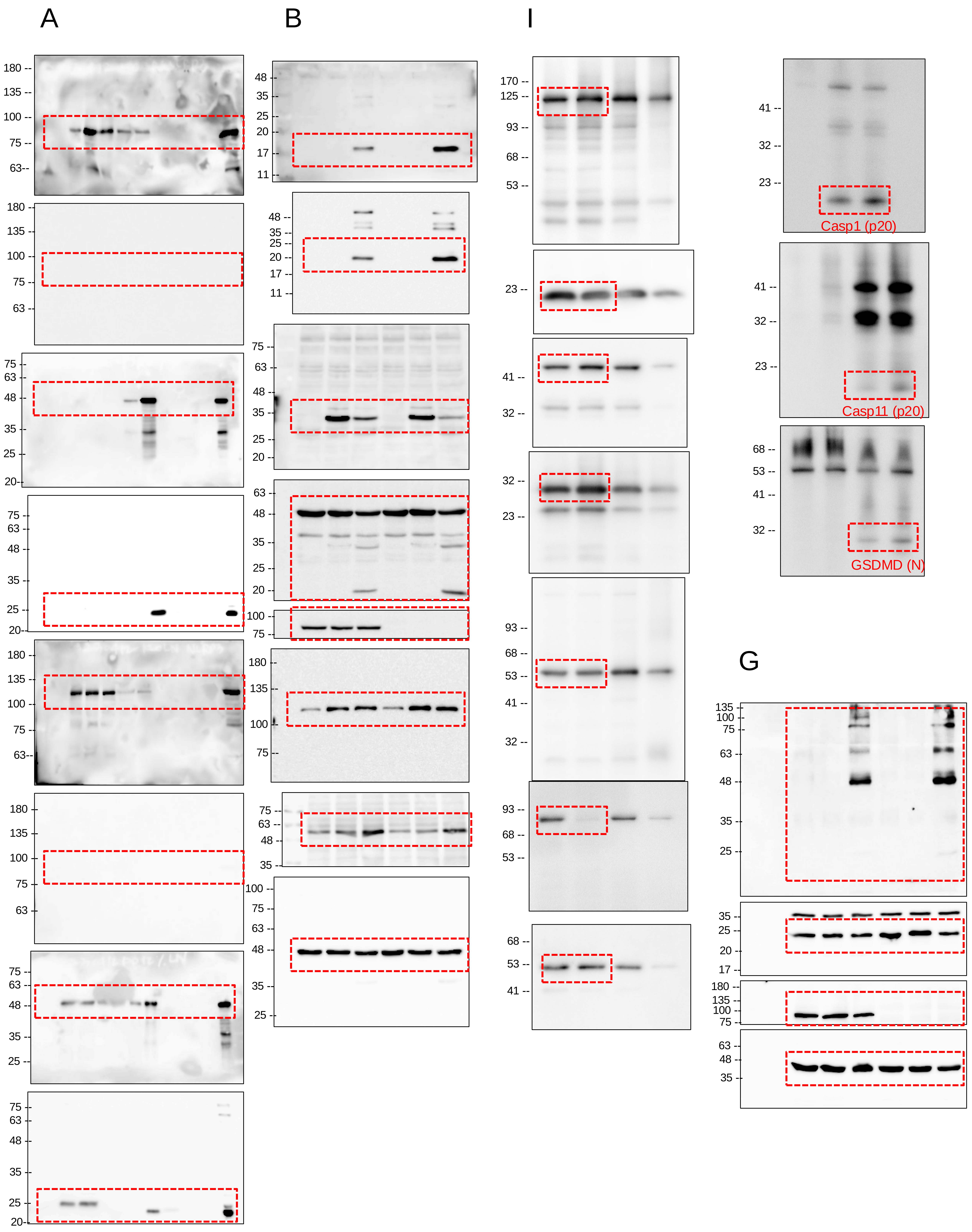

Supplementary Figure 4

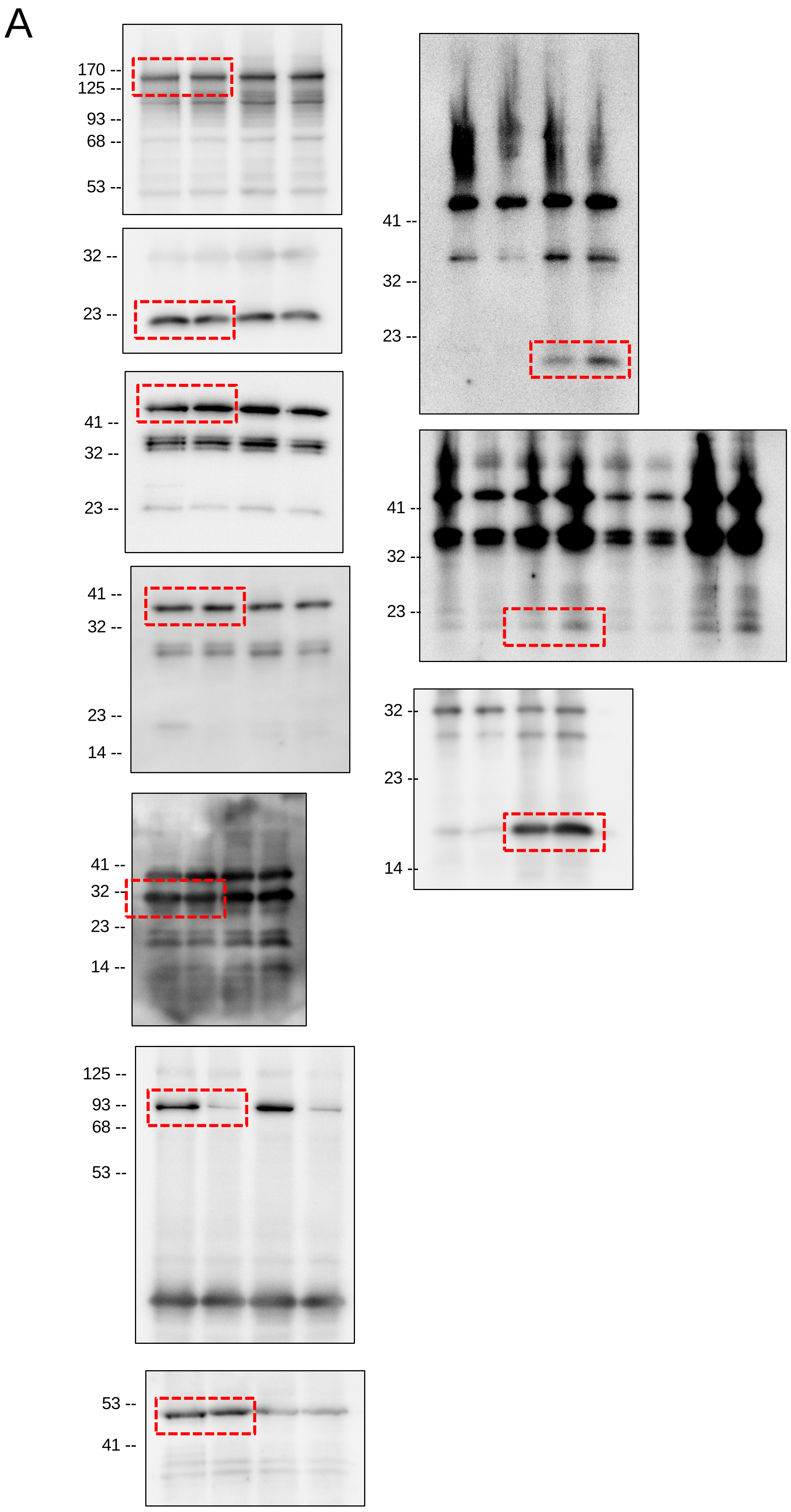

Supplementary Figure 5

D

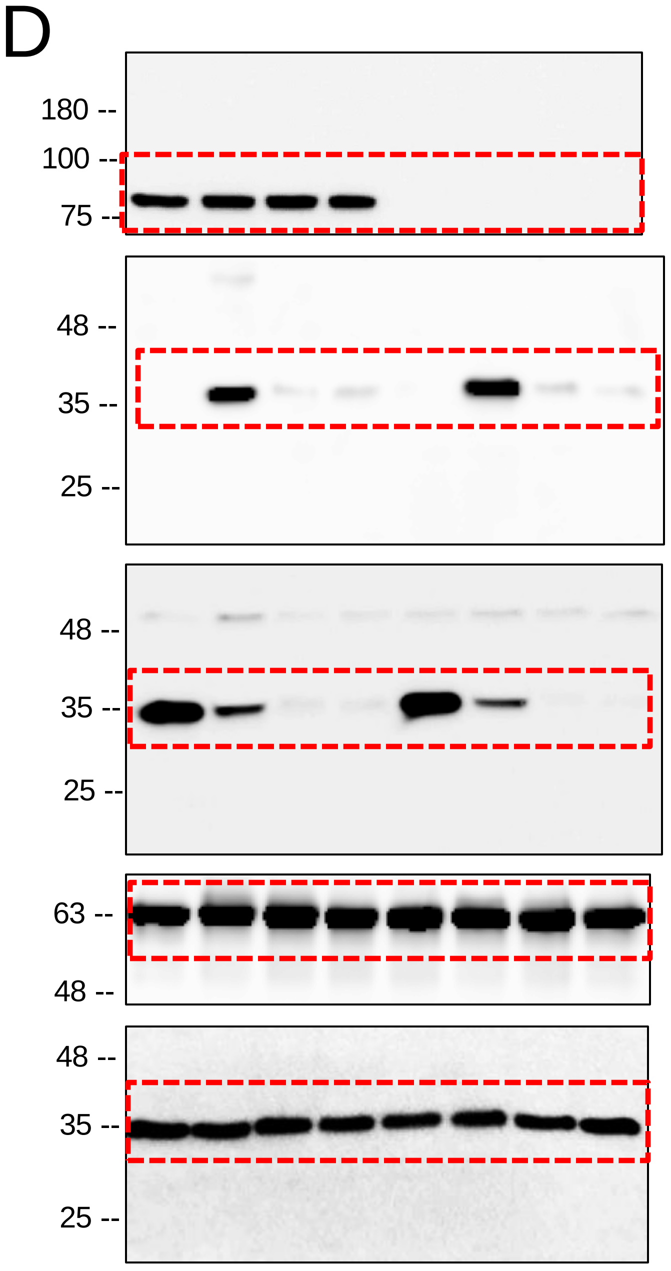

Supplementary Figure 6

A

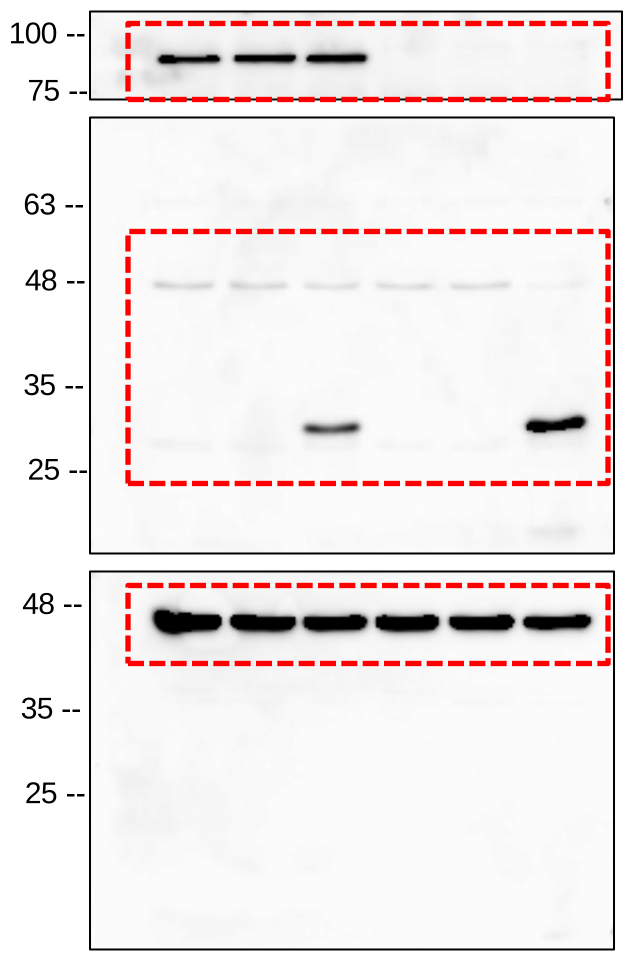

Supplementary Figure 9

C

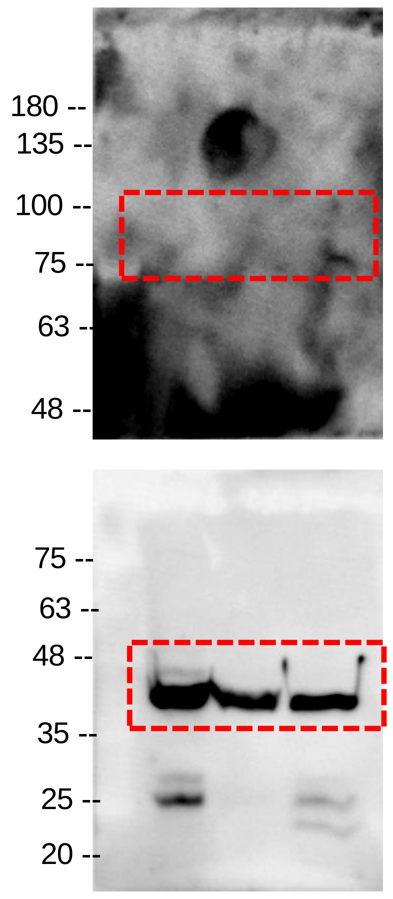

Supplement: Supplementary file 2 — Uncut Images for Figures [file 41418_2024_1367_MOESM2_ESM.pdf]
